# A first generation integrated map of the rainbow trout genome

**DOI:** 10.1186/1471-2164-12-180

**Published:** 2011-04-07

**Authors:** Yniv Palti, Carine Genet, Ming-Cheng Luo, Aurélie Charlet, Guangtu Gao, Yuqin Hu, Cecilia Castaño-Sánchez, Kamila Tabet-Canale, Francine Krieg, Jianbo Yao, Roger L Vallejo, Caird E Rexroad

**Affiliations:** 1 National Center for Cool and Cold Water Aquaculture, ARS-USDA, 11861 Leetwon Road, Kearneysville, WV 25430, USA; 2 INRA, UMR1313, Génétique Animale et Biologie Intégrative, Domaine de Vilvert, 78352 Jouy en Josas Cedex, France; 3 Department of Plant Sciences, University of California, One Shields Ave., Davis, CA 95616, USA; 4 West Virginia University, Animal and Nutritional Sciences, Morgantown, WV, 26506, USA; 5 INRA, UMR 444 ENVT Génétique Cellulaire, 31326 Castanet-Tolosan, France

## Abstract

**Background:**

Rainbow trout (*Oncorhynchus mykiss*) are the most-widely cultivated cold freshwater fish in the world and an important model species for many research areas. Coupling great interest in this species as a research model with the need for genetic improvement of aquaculture production efficiency traits justifies the continued development of genomics research resources. Many quantitative trait loci (QTL) have been identified for production and life-history traits in rainbow trout. An integrated physical and genetic map is needed to facilitate fine mapping of QTL and the selection of positional candidate genes for incorporation in marker-assisted selection (MAS) programs for improving rainbow trout aquaculture production.

**Results:**

The first generation integrated map of the rainbow trout genome is composed of 238 BAC contigs anchored to chromosomes of the genetic map. It covers more than 10% of the genome across segments from all 29 chromosomes. Anchoring of 203 contigs to chromosomes of the National Center for Cool and Cold Water Aquaculture (NCCCWA) genetic map was achieved through mapping of 288 genetic markers derived from BAC end sequences (BES), screening of the BAC library with previously mapped markers and matching of SNPs with BES reads. In addition, 35 contigs were anchored to linkage groups of the INRA (French National Institute of Agricultural Research) genetic map through markers that were not informative for linkage analysis in the NCCCWA mapping panel. The ratio of physical to genetic linkage distances varied substantially among chromosomes and BAC contigs with an average of 3,033 Kb/cM.

**Conclusions:**

The integrated map described here provides a framework for a robust composite genome map for rainbow trout. This resource is needed for genomic analyses in this research model and economically important species and will facilitate comparative genome mapping with other salmonids and with model fish species. This resource will also facilitate efforts to assemble a whole-genome reference sequence for rainbow trout.

## Background

Rainbow trout (*Oncorhynchus mykiss*) are the most-widely cultivated cold freshwater fish in the world and are considered by many to be the "aquatic lab-rat". Interests in the utilization of rainbow trout as a model species for genome-related research activities focusing on carcinogenesis, toxicology, comparative immunology, disease ecology, physiology, transgenics, evolutionary genetics, and nutrition have been well documented [1]. Rainbow trout are cultured on every continent except Antarctica, with 2008 global production estimated at 576,289 metric tons and valued at $2.39 billion [2]. Coupling great interest in this species as a research model with the need for genetic improvement for aquaculture production efficiency and product quality justifies the continued development of genome resources facilitating selective breeding.

The rainbow trout genome is large and complex. Genome size estimates derived from determining the molecular weight of DNA per cell for rainbow trout and other salmonids vary from 2.4 to 3.0 × 10^9 ^bp [3,4]. As with most salmonids, rainbow trout experienced a recent genome duplication event resulting in a semi-tetraploid state (i.e. after an autotetraploid event in the salmonids, their genome is undergoing reversion to a diploid state) [5]. All ray-finned fishes share an additional (3R) round of ancestral genome duplication in their evolutionary history compared to mammals and birds, but the salmonids' common ancestor underwent an additional recent (4R) whole genome duplication event and more than half of the loci are still duplicated [6]. In addition, it is estimated that 50% to 60% of the rainbow trout genome contains interspersed repeat sequences (Genet et al.: Analysis of BAC-end sequences in rainbow trout: content characterization and assessment of synteny between trout and other fish genomes, submitted).

Current genomic resources available for rainbow trout research include multiple bacterial artificial chromosome (BAC) libraries and a BAC fingerprinting physical map [6-8]; a database of ~200,000 BAC end sequences (BES) (Genet et al.: Analysis of BAC-end sequences in rainbow trout: content characterization and assessment of synteny between trout and other fish genomes, submitted); doubled haploid (DH) clonal lines [9-12]; multiple genetic maps based on clonal lines and outbred populations [4,13-16]; large expressed sequence tag (EST) databases and a reference transcriptome [17-19]; a microRNAs database [20] and high density DNA microarrays [21,22].

Two microsatellite-based genetic maps with medium to high marker densities were recently developed for rainbow trout by INRA [13] and the NCCCWA [16]. The INRA map is based on a panel of two DH gynogenetic lines. It has more than 900 microsatellites over 31 linkage groups and a total length of 2,750 cM (average resolution of 3 cM). The NCCCWA map is based on a panel of five families that represent the starting genetic material of the NCCCWA selective breeding program. It has 1,124 microsatellite loci over 29 linkage groups and a total length of 2,927 cM (average resolution of 2.6 cM). The linkage groups from the two microsatellite genetic maps were anchored to the physical chromosomes using fluorescent in-situ hybridization and were found to represent 52 chromosome arms [23,24].

Qualitative/quantitative trait loci (QTL) mapping experiments in rainbow trout have been very successful because of their high fecundity, external fertilization, and ease of gamete handling and manipulation. Many QTL have been identified for production and life-history traits including resistance to the parasite *C. shasta *[25], resistance to IHNV [26,27] and to IPNV [28], whirling disease resistance [29], Killer cell-like activity [30], upper thermal tolerance [31,32], embryonic development rate [9,33,34], spawning time [35,36], confinement stress response [37], early maturation [38] and smoltification [39]. The availability of a BAC physical map integrated with the genetic map will facilitate fine mapping of QTL, the selection of positional candidate genes and the incorporation of marker-assisted selection (MAS) into rainbow trout breeding programs. A major shortcoming of QTL studies is that they are limited to the variation present in a limited number of families and typically do not detect loci with small effects. This can be overcome by whole genome association studies and other approaches, such as genomic selection, that capture the effects of most QTL that contribute to the population-wide variation in a trait. Recently we demonstrated the feasibility of low resolution LD association studies in rainbow trout [40,41]. In the absence of a reference genome sequence assembly, a robust integrated physical and genetic map will provide better resolution than the current genetic maps for ordering of genetic markers and estimating physical distances between markers, thus facilitating future whole genome association studies in rainbow trout.

The first BAC-based physical map of the rainbow trout genome was recently assembled using DNA fingerprints of 154,439 clones from the 10X HindIII Swanson library [8]. The map contains 4,173 contigs and 9,379 singletons. The physical length of the map contigs was estimated to be approximately 2.0 Gb, which represents approximately 80% of rainbow trout genome. Here we report the construction of the first integrated physical and genetic map of the rainbow trout genome using microsatellites isolated from BAC end sequences and PCR superpools for library screening and identification of BACs that harbor previously mapped markers. This integrated map provides a frame work for a robust composite genome map and future reference genome sequence assemblies.

## Results and Discussion

### BAC end sequencing (BES) microsatellites

We screened the BES reads from 184 of the largest BAC fingerprinting contigs and selected 205 microsatellites from 117 contigs for PCR optimization and genotyping (Table 1). Of the 205 markers genotyped, 128 markers appeared to amplify single marker regions and were polymorphic. Ten markers were monomorphic, and 58 markers could not be resolved and unambiguously scored. Fifteen markers generated duplicated patterns, of which 8 could be scored for a single marker region and 1 produced a scorable duplicated pattern. Hence, 7 of the duplicated markers produced a monomorphic or an unresolved pattern for one of the two marker regions. Two of the 128 informative markers could not be assigned to linkage groups (i.e. 126 markers were mapped using the NCCCWA mapping families). The BES reads from which the 126 mapped markers were isolated represent 88 unique BAC FPC contigs. The 205 BES microsatellites are listed in Additional file 1, sheet 1, with the corresponding PCR primers and conditions for each marker, number of alleles and size range, GenBank accessions, primers sequences and physical map contigs. We have also mapped an additional six BES microsatellites onto linkage groups of the INRA genetic map (Additional file 1, sheet 1).

**Table 1:** Summary of genotyping results of microsatellite markers isolated from BAC end sequences for integration between the genetic and physical maps

No. of markers identified	205 (from 117 contigs)
Informative for linkage analysis	128 (63%; 88 contigs; 129 loci)
Mapped to linkage groups	127 loci (98.5%; 88 contigs)
PCR optimization failed	58 (28%)
Monomorphic in mapping panel	10 (5%)
Duplicated	15 (9 informative for mapping and 6 non-informative)
Redundancy in contig coverage (optimized, but panel not genotyped)	3 markers

### Library screening with PCR superpools

#### Previously mapped microsatellites

The 10x Swanson BAC library was screened with the NCCCWA PCR super-pools using 137 markers that were previously mapped with high confidence to the NCCCWA genetic map representing 25 of the 29 chromosomes and the INRA super-pools were screened with 265 markers that were previously mapped onto the INRA genetic map representing all linkage groups. The result of the combined effort was that 146 markers covering all linkage groups were localized to one or two BAC FPC contigs (Table 2). The list of the markers with positive hits is shown in Additional file 1, sheet 2, with the corresponding positive clones and physical map contigs.

**Table 2:** Summary of BAC library screening results with previously mapped microsatellites using PCR super-pools

	**INRA**^**a**^	**USDA**^**b**^	Combined
No. of markers tested	265	137	396
Localized to a single FPC contig	98	41	135
Localized to two FPC contigs	7	5	11
Singletons or failed DNA fingerprinting	21	15	35
Not validated by single clone PCR	4	12	16
Not positive by PCR screening of superpools	135	64	199
No. of chromosomes covered	29	22	29

^a ^Markers previously mapped on the INRA genetic map [13].

^b ^Markers previously mapped on the USDA-NCCCWA genetic map [16].

#### Immune response genes

The BAC library was also screened with PCR primers from 12 immune response genes that were not previously mapped to the rainbow trout genome (Additional file 2, Table S1). Positive clones were verified by PCR of the individual clones and direct sequencing from the BAC DNA. The BAC clones that were positive and their corresponding physical map contigs are listed in Additional file 1, sheet 3.

### Single nucleotide polymorphism (SNP) markers

The experimental design and results of SNPs discovery in rainbow trout using a reduced representation library (RRL) were recently published [42]. Of the 183 SNPs that were validated, 167 were polymorphic in the NCCCWA genetic mapping panel and 159 were mapped to chromosomes on the genetic map (Table 3). The HaeIII RRL SNP discovery database was aligned with the BES database (Genet et al.: Analysis of BAC-end sequences in rainbow trout: content characterization and assessment of synteny between trout and other fish genomes, submitted) to find matches that can be useful for the integration of the genetic and physical maps. We found 618 unique matches using SSAHA2 [43]. Assuming 48% validation rate for this SNPs database [42] we expect that approximately 300 of the matched SNPs will be useful for integration between the physical and genetic maps. Two of the matching SNPs were among the 183 validated by Castaño-Sánchez et al. [42]. One marker (OMS00144) was among the 159 that were mapped. The other SNP (OMS00174) was not informative for linkage analysis in the NCCCWA panel, but it had two positive hits on end sequences from two BACs that overlap in contig number 431 of the physical map (Additional file 1, sheet 3).

**Table 3:** Genetic loci sources and linkage mapping statistics

Marker Source	**Input**^**a**^	Mapped	LOD4	LOD3	LOD2	LOD1	LOD0	% of Input
Rexroad et al. 2008 [16]	1180	1126^b^	396	62	57	43	568	95%
SNPs (OMS)	167	159	21	5	13	8	112	95%
OMY4000 (BES)	128	127	40	10	10	5	62	98%
Immune Genes^c^	10	9	3	0	0	0	6	90%
Total	1485	1421^d^	460	77	80	56	748	96%
Percent		100%	32%	5%	6%	4%	53%	

^a ^Loci that were genotyped and were informative for linkage analysis in the NCCCWA mapping panel.

^b ^Twenty seven loci that were previously mapped to linkage groups in the genetic map version of Rexroad et al. (2008) were not linked to other loci in this version, and 29 loci that were previously genotyped but not linked were assigned to linkage groups in the current version.

^c ^Microsatellites or SNPs isolated from immune response genes (Additional file 2, Table S2).

^d ^Eighty eight duplicated loci were mapped to linkage groups in the current version (total number of markers was 1,333).

### The genetic map

**Figure 1:**
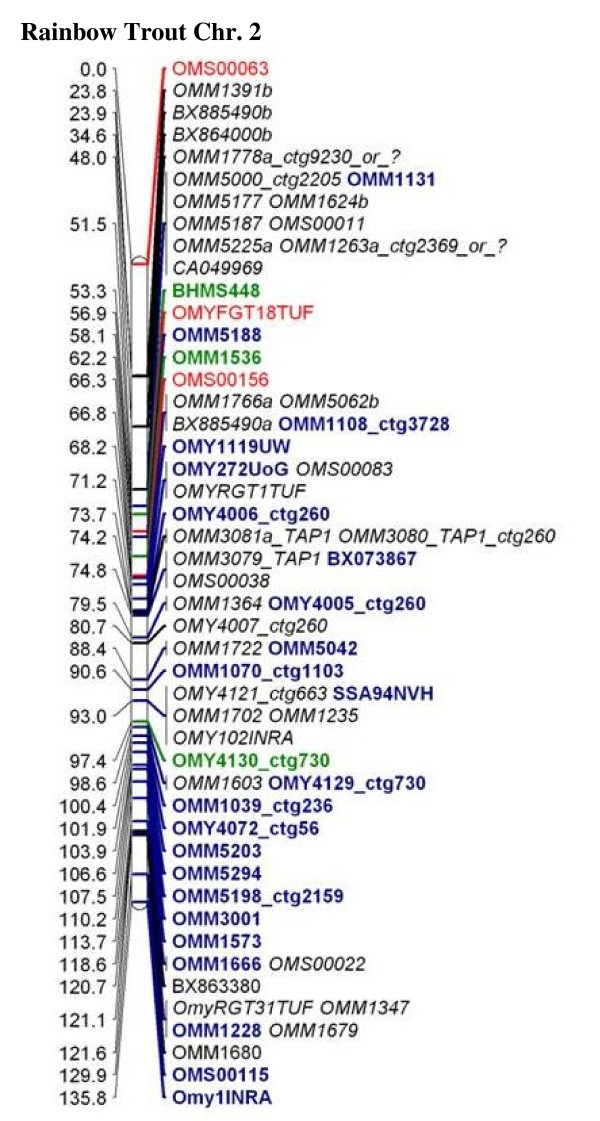
**Chromosome 2 from the new NCCCWA linkage map is shown as an example**. Annotation of genes linked to the marker or BAC contig from the 1^st ^generation physical map are connected to the marker name by underscore (e.g. OMM3080_TAP1_ctg260). Annotation of "or_?" means that the marker is duplicated and only one of two BAC contig was identified for the marker. Blue, Green, Red, Black and Italicized font markers were mapped to their specific location on the linkage group at LOD scores of 4, 3, 2, 1 and 0, respectively. Sex average distances between markers are shown in cM.

Information from 1,486 genetic loci was used for linkage analysis (Table 3). Two-point linkage analysis placed 1,229 loci in 29 linkage groups at LOD ≥8.75. An additional 192 markers with two-point LOD <8.75 were added to linkage groups manually, of which only six markers had a two-point LOD <3.0 (2.90, 2.89, 2.64, 2.12, 2.10 and 1.80). The specific best of two-point LOD score for each marker is provided in Additional file 3, Worksheet 1. The total combined sex averaged map distance was 3,346.3 cM (Kosambi). A sample map representing chromosome 2 is presented in Figure 1, and maps representing all chromosomes are presented in Additional file 4. Multipoint linkage analysis was conducted on individual linkage groups to assign LOD scores for the specific position of each marker within the linkage group. The number of markers included in a framework map created at LOD ≥4 for the specific position of the marker in the linkage group was 460. The only chromosome that did not contain any framework markers at LOD ≥4 was OMY21, for which a framework map was created at LOD ≥3. Additional loci were added at LOD ≥3 (77), ≥2 (80) ≥1 (56), and ≥0 (748) (Table 3). Additional file 3, worksheet 1 contains this information and can be used to recreate maps using MapChart software [44]. The average resolution of the genetic map was 2.35 cM with inter-marker distances ranging from 1.31 to 3.59 cM for individual chromosomes (Additional file 3, Worksheet 2).

The female:male recombination ratio was 1.65:1, with the female having a map length of 4,775.7 cM and the male map 2,897.8 cM. This ratio varied by chromosome, ranging from 0.53:1 to 11.87:1 (Additional file 3, Worksheet 2). It is noteworthy that this type of sex recombination ratio estimates do not take into account the larger differences in recombination rate that exist between males and females throughout most of the length of the linkage groups. It is likely that female:male ratios will be elevated throughout most of the length of the chromosome arms, while they will be much lower in the more contracted telomeric ends of the linkage groups because of elevated male recombination rates in these regions [15]. It should be pointed out that overall estimates of recombination rate may not be accurately depicted in the current study, because recombination estimates were not obtained by direct comparisons of adjacent intervals. Therefore, the reported recombination distances given in this study are likely an underestimate of the real recombination ratio values.

In this version of the map, we have added to the map of Rexroad et al. [16] through multipoint linkage analysis 159 RRL SNPs, 126 microsatellites from BES and 9 microsatellites isolated from BACs that harbor immune response genes (Additional file 2, Table S2). The SNPs were distributed in all the chromosomes (2-10 per chromosome; Additional file 3, worksheet 3) and the BES microsatellites were mapped to all but chromosome 24 (1-10 per chromosome; Additional file 3, worksheet 4). Twenty seven loci that were previously mapped to expand the length of linkage groups [16] were not mapped in this version, and 29 loci that were previously genotyped but were not linked, were assigned to linkage groups in the current version. A high frequency of duplicated microsatellite loci was observed as previously reported [16], but in many cases only one locus was successfully ordered on the map. Overall, 88 duplicated markers were successfully mapped to two loci (176 loci), which means that the total number of markers mapped was 1,333.

### The integrated map

**Figure 2:**
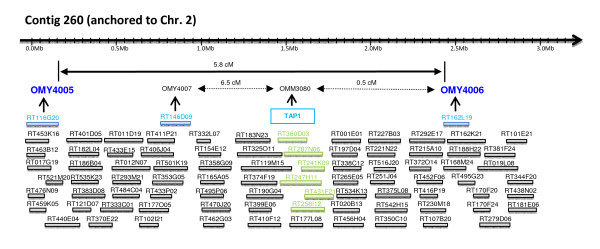
**A schematic illustration of a BAC fingerprinting contig anchored to the rainbow trout Chr. 2 using microsatellites isolated from BACs**. The four microsatellite markers from Ctg260 (224 clones; 1,584 CB or approximately 2.7 Mb) were mapped to Chr. 2 and the TAP1 positive BACs (highlighted in green) were previously identified by probe hybridization and confirmed by PCR and direct sequencing. The microsatellites order shown is based on the FPC map (not the genetic map). Markers in bold blue (OMY4005 and 4006) were localized on the linkage group at LOD4 and markers in regular font at LOD0. The genetic distance between the LOD4 markers is marked by a solid-line arrow and between markers that were localized at lower confidence by broken-line arrows.

Anchoring of 203 BAC contigs from the physical map to linkage groups was accomplished through mapping of 266 loci onto the NCCCWA genetic map. The marker loci were derived from the PCR screening of the BAC superpools, BES microsatellites (OMY4000), microsatellites isolated from BACs that harbor genes of interest (OMM3000) and one SNP marker matched with BES (OMS00144). A schematic illustration of a BAC fingerprinting contig anchored to a linkage group is presented in Figure 2. Markers from 12 of the anchored contigs were mapped to two different linkage groups as a result of PFC assembly errors or linkage mapping errors as we have previously discussed [8]. The fraction of contigs that are in disagreement between the physical map and genetic map is used to estimate the error rate in the FPC assembly. This error rate of 6% (12/203) is similar to the 5% estimated for the catfish physical map of Quiniou et al. [45] or the 4% rate detected in the 3-color HICF physical map of the maize genome [46]. The number of contigs anchored per chromosome ranged from 3 to 17 with an average of 7.4. Chromosomes OMY18, 24 and 28 had the lowest number of 3 anchored contigs each, and OMY12 had the highest number with 17 anchored contigs.

The combined physical length of the 203 anchored contigs was 138,525 consensus bands (CB) which is equal to 235,493 Kb based on a conversion ratio of 1 CB = 1.7 Kb [8]. Therefore, we estimate that the integrated map covers ~12% of the physical map, or ~10% of the rainbow trout genome, assuming haploid genome size of 2.4 × 10^9 ^bp. The length of anchored contigs ranged from 119 Kb to 4,590 Kb with an average length of 1,160 Kb (Additional file 3, worksheet 5). The ratio of physical to genetic linkage distances varied substantially among the 33 anchored contigs that contained spaced markers, which is similar to other vertebrate genomes [45,47]. The 33 contigs represent segments from 21 of the 29 chromosomes (Additional file 3, worksheet 6). The Kb/cM ratio ranged from 37 to 17,000 with an average of 3,033. In addition, 35 contigs were anchored to linkage groups of the INRA map through markers that were not informative for linkage analysis in the NCCCWA mapping panel (Additional file 3, worksheet 7), bringing the total number of anchored contigs to 238.

An FPC map with all the genetic markers that we have assigned to BAC contigs can be viewed and searched online through: http://www.genome.clemson.edu/activities/projects/rainbowTrout

The integrated map we developed for the rainbow trout genome will facilitate comparative genomics studies with other salmonids and with model fish species. Many microsatellite markers can be used for genetic mapping across salmonid species which is very useful for comparative genome mapping [23,48] and can benefit research in species with less developed genome maps. In addition, the rainbow trout BAC end sequences can be used to infer conserved synteny with other fish genomes as we have previously shown (Genet et al.: Analysis of BAC-end sequences in rainbow trout: content characterization and assessment of synteny between trout and other fish genomes, submitted), and this integrated map provides a larger frame-work expanding the size of the syntenic blocks that can be identified between fish genomes.

## Conclusions

The first generation integrated map of the rainbow trout genome is composed of 238 BAC contigs anchored to chromosomes of the genetic map. It covers more than 10% of the genome across segments from all 29 chromosomes. This map provides a frame work for a robust composite genome map. The availability of an integrated physical and genetic map will enable detailed comparative genome analyses, fine mapping of QTL, positional cloning, selection of positional candidate genes for economically important traits and the incorporation of MAS into rainbow trout breeding programs. A comprehensive integrated map will also provide a minimal tiling path for genome sequencing and a framework for whole genome sequence assembly.

## Methods

### BAC end sequencing and markers development

The 10X HindIII Rainbow trout BAC library [6] was used for BAC-end sequencing (BES) as previously described (Genet et al.: Analysis of BAC-end sequences in rainbow trout: content characterization and assessment of synteny between trout and other fish genomes, submitted). Briefly, BAC culture was conducted using standard protocols and end sequencing with SP6 and T7 primers was done using standard Sanger technique. The raw, untrimmed files were processed by PHRED software [49]. The PHRED quality score cut-off value was set at 20 for the acquisition of Q20 values. The BESs were trimmed of vector sequences (pBeloBAC11 vector [50]) and filtered of *E. coli *sequences. Microsatellites and other simple sequence repeats (SSR) were analyzed using Tandem repeat Finder software [51]. We examined ten classes of SSRs by using a maximum period size of 10. BES reads harboring at least 50 base pairs (bp) flanking sequences on either side of the SSRs were selected for PCR primer design. Primers for BESs containing microsatellites were designed using Primer3 software [52]. The primer product size range was chosen between 150 and 450 nucleotides. The optimum size of primers was set to 20 nucleotides (range from 18 to 27 nucleotides) with an optimum melting temperature of 60.0°C (range from 57 to 63°C).

### Microsatellites Genotyping

The NCCCWA mapping panel of 5 families was genotyped with microsatellites as previously described [16]. A total of 205 microsatellite markers isolated from BAC end sequences (Additional file 1, sheet 1) were genotyped using the tailed protocol of Boutin-Ganache *et al. *[53]. Primers were obtained from commercial sources (Alpha DNA, Montreal, Quebec, Canada). Three oligonucleotide primers were used in each DNA amplification reaction (Forward: 5' GAGTTTTCCCAGTCACGAC-primer sequence 3'; reverse: 5' GTTT-primer sequence 3'; fluorescent labeled primer with FAM: 5' GAGTTTTCCCAGTCACGAC 3'). Primers were optimized for amplification by varying annealing temperatures and MgCl2 concentrations. PCR reactions (12 μl total volume) included 50 ng DNA, 1.5-2.5 mM MgCl_2_, 2 pmol of forward primer, 6 pmol of reverse primer, 1 pmol of fluorescent labeled primer, 200 μM dNTPs, 1X manufacturer's reaction buffer, and 0.5 unit Taq Polymerase (ABI, Foster City, CA, USA). Amplifications were conducted in an MJ Research DNA Engine thermal cycler model PTC 200 (MJ Research, Waltham, MA) as follows: an initial denaturation at 95°C for 10 min, 30 cycles consisting of 94°C for 60 sec, annealing temperature for 45 sec, 72°C extension for 45 sec; followed by a final extension of 72°C for 10 min. PCR products were visualized on agarose gels after staining with ethidium bromide. Three μl of each PCR product was added to 20 μl of water, 1 μl of the diluted sample was added to 12.5 μl of loading mixture made up with 12 μl of HiDi formamide and 0.5 μl of Genscan 400 ROX internal size standard. Samples were denatured at 95°C for 5 min and kept on ice until loading on an ABI 3730 DNA Analyzer (ABI, Foster City, CA, USA). Output files were analyzed using GeneMapper version 3.7 (ABI, Foster City, CA, USA), formatted using Microsoft Excel and stored in a Microsoft Access database.

### Library screening with PCR superpools

The 10x Swanson BAC library was screened using the NCCCWA or the INRA PCR superpools with microsatellites that were mapped to the NCCCWA or INRA genetic maps [13,16] as previously described [54,55]. The screening results were cross-referenced with the physical map to localize the positive clones onto contigs. For microsatellite markers that did not have at least two positive clones from the same FPC BAC contig, the individual positive clones were picked from glycerol stock and confirmed by PCR as previously described [6].

### SNPs discovery using reduced representation libraries (RRL)

Protocols developed and used for SNPs discovery in cattle and swine [56-58] were adapted for rainbow trout using RRL libraries and high throughput parallel 454 GS FLX pyrosequencing. The experimental design and results of the rainbow trout work were recently published [42]. Briefly, DNA from 96 unrelated individuals representing the families of the NCCCWA broodstock was pooled into one sample. The reduced representation library consisted of 440 bp fragments resulting from complete digestion of the pooled DNA with the restriction enzyme *Hae*III; sequencing produced 2,000,000 reads providing an average 6 fold coverage of the estimated 150,000 unique genomic restriction fragments (300,000 fragment ends). Three independent computational data analyses identified 22,022 to 47,128 putative SNPs on 13,140 to 24,627 contigs. A set of 384 putative SNPs, randomly selected from the sets produced by the three analyses were genotyped on individual fish to determine the validation rate of putative SNPs among analyses, distinguish apparent SNPs that actually represent paralogous loci in the semi-tetraploid genome, examine Mendelian segregation, and place the validated SNPs on the rainbow trout linkage map.

### Alignments between SNPs and BES

To find matches we aligned the HaeIII RRL SNP discovery database of Castaño-Sánchez et al. [42] with the BES database (Genet et al.: Analysis of BAC-end sequences in rainbow trout: content characterization and assessment of synteny between trout and other fish genomes, submitted). Matches were found using SSAHA2 [43] (http://www.sanger.ac.uk/Software/analysis/SSAHA2/) for pairwise sequence alignment with a threshold Smith-Waterman score of 160 (very restrictive and conserved to avoid matches between paralogous loci).

### Linkage analysis

The microsatellites and SNPs were placed on the rainbow trout genetic map using the genetic linkage mapping programs MULTIMAP [59] and CRI-MAP [60]. First, genotype data combined for both sexes were formatted into the standard LINKAGE [61] file format and checked for Mendelian inheritance using PEDCHECK [62]. RECODE [63] was then used to convert the allele sizes into number-coded alleles. Using an in-house Perl script, make_gen, the genotype data and the locus names were assembled into CRI-MAP input format. The resulting file was then added to that of Rexroad *et al. *[16] using another in-house Perl script, join_gens, and MULTIMAP was used to conduct two-point linkage analyses to identify the closest markers with LOD ≥8.75 and recombination fraction r ≤0.2. An additional 192 markers with two-point LOD <8.75 were added to linkage groups manually, of which only six markers had a two-point LOD <3.0 (2.90, 2.89, 2.64, 2.12, 2.10 and 1.80). The specific best of two-point LOD score for each marker is provided in Additional File 3, Worksheet 1. Multipoint linkage analysis was conducted on individual linkage groups to assign LOD scores for the specific position of each marker within the linkage group. Framework maps were constructed at LOD ≥4 for all linkage groups but OMY21, for which the framework map was created at LOD ≥3. Markers were added to comprehensive maps by lowering the LOD threshold one integer at a time and starting with the previous order. Resulting maps are consensus maps, accounting for co-informative meiosis across the five families. Chromosome numbers were assigned to linkage groups using the integrated cytogenetic/linkage map of Phillips *et al. *[24].

## Authors' contributions

 conceived and designed the study, supervised and analyzed microsatellites genotyping, supervised the USDA BAC library screening, integrated the markers information from the genetic and physical maps, updated the physical map and wrote the manuscript draft; CG participated in the study design, obtained BAC end sequences, identified microsatellites in BAC end sequences and designed PCR primers for microsatellites genotyping and supervised the INRA BAC library screening; MCL participated in the study design, supervised DNA extractions and BAC fingerprinting and assembled the physical map; AC screened the INRA PCR superpools; GG improved the genetic linkage analysis pipeline, conducted the linkage analysis and assembled the genetic map, and conducted the SSAHA2 alignment between the SNP and BES databases; YH conducted DNA extractions and BAC fingerprinting; KTA contributed to the INRA BAC library pooling and screening; FK contributed to the INRA BAC library screening; CCS conducted the SNPs discovery and validation experiments; JY co-supervised the SNPs discovery and validation experiments; RLV participated in the study design and developed the genetic linkage analysis pipeline; CER participated in the study design and in the genetic linkage analysis and supervised the SNPs discovery and validation experiments. All authors reviewed and contributed to the manuscript.

## Supplementary Material

Additional file 1 BES microsatellites Click here for file

Additional file 2 Table S1 Click here for file

Additional file 3 Additional material Click here for file

Additional file 4 chromosome maps Click here for file

## References

[B1] Thorgaard GH Bailey GS Williams D Buhler DR Kaattari SL Ristow SS Hansen JD Winton JR Bartholomew JL Nagler JJ Status and opportunities for genomics research with rainbow trout Comp Biochem Physiol B Biochem Mol Biol 2002 133 4 609 646 10.1016/S1096-4959(02)00167-7 12470823

[B2] FAO Cultured Aquatic Species Information Programme. Oncorhynchus mykiss. FAO Fisheries and Aquaculture Department 2010 http://www.fao.org/fishery/culturedspecies/Oncorhynchus_mykiss/en

[B3] Ng SH Artieri CG Bosdet IE Chiu R Danzmann RG Davidson WS Ferguson MM Fjell CD Hoyheim B Jones SJ A physical map of the genome of Atlantic salmon, Salmo salar Genomics 2005 86 4 396 404 10.1016/j.ygeno.2005.06.001 16026963

[B4] Young WP Wheeler PA Coryell VH Keim P Thorgaard GH A detailed linkage map of rainbow trout produced using doubled haploids Genetics 1998 148 2 839 850 9504929 10.1093/genetics/148.2.839 PMC1459819

[B5] Allendorf FW Thorgaard GH Turner BJ Tetraploidy and the evolution of salmonid fishes Evolutionary Genetics of Fishes 1984 New York: Plenum Press 1 46

[B6] Palti Y Gahr SA Hansen JD Rexroad CE Characterization of a new BAC library for rainbow trout: evidence for multi-locus duplication Anim Genet 2004 35 2 130 133 10.1111/j.1365-2052.2004.01112.x 15025574

[B7] Katagiri T Asakawa S Minagawa S Shimizu N Hirono I Aoki T Construction and characterization of BAC libraries for three fish species; rainbow trout, carp and tilapia Anim Genet 2001 32 4 200 204 10.1046/j.1365-2052.2001.00764.x 11531698

[B8] Palti Y Luo MC Hu Y Genet C You F Vallejo R Thorgaard G Wheeler P Rexroad C A first generation BAC-based physical map of the rainbow trout genome BMC Genomics 2009 10 1 462 10.1186/1471-2164-10-462 19814815 PMC2763887

[B9] Robison BD Wheeler PA Sundin K Sikka P Thorgaard GH Composite interval mapping reveals a major locus influencing embryonic development rate in rainbow trout (Oncorhynchus mykiss) J Hered 2001 92 1 16 22 10.1093/jhered/92.1.16 11336224

[B10] Robison BD Wheeler PA Thorgaard GH Variation in development rate among clonal lines of rainbow trout (Oncorhynchus mykiss) Aquaculture 1999 173 1-4 131 141 10.1016/S0044-8486(98)00481-5

[B11] Young WP Wheeler PA Fields RD Thorgaard GH DNA fingerprinting confirms isogenicity of androgenetically derived rainbow trout lines J Hered 1996 87 1 77 80 8742822 10.1093/oxfordjournals.jhered.a022960

[B12] Quillet E Dorson M Le Guillou S Benmansour A Boudinot P Wide range of susceptibility to rhabdoviruses in homozygous clones of rainbow trout Fish & Shellfish Immunology 2007 22 5 510 519 10.1016/j.fsi.2006.07.002 17085058

[B13] Guyomard R Mauger S Tabet-Canale K Martineau S Genet C Krieg F Quillet E A type I and type II microsatellite linkage map of rainbow trout (Oncorhynchus mykiss) with presumptive coverage of all chromosome arms BMC Genomics 2006 7 302 10.1186/1471-2164-7-302 17137492 PMC1698487

[B14] Nichols KM Young WP Danzmann RG Robison BD Rexroad C Noakes M Phillips RB Bentzen P Spies I Knudsen K A consolidated linkage map for rainbow trout (Oncorhynchus mykiss) Anim Genet 2003 34 102 115 10.1046/j.1365-2052.2003.00957.x 12648093

[B15] Sakamoto T Danzmann RG Gharbi K Howard P Ozaki A Khoo SK Woram RA Okamoto N Ferguson MM Holm L-E A Microsatellite Linkage Map of Rainbow Trout (Oncorhynchus mykiss) Characterized by Large Sex-Specific Differences in Recombination Rates Genetics 2000 155 3 1331 1345 10880492 10.1093/genetics/155.3.1331 PMC1461176

[B16] Rexroad CE Palti Y Gahr SA Vallejo RL A second generation genetic map for rainbow trout (Oncorhynchus mykiss) BMC Genet 2008 9 74 10.1186/1471-2156-9-74 19019240 PMC2605456

[B17] Rexroad CE Lee Y Keele JW Karamycheva S Brown G Koop B Gahr SA Palti Y Quackenbush J Sequence analysis of a rainbow trout cDNA library and creation of a gene index Cytogenet Genome Res 2003 102 1-4 347 354 10.1159/000075773 14970727

[B18] Govoroun M Le Gac F Guiguen Y Generation of a large scale repertoire of Expressed Sequence Tags (ESTs) from normalised rainbow trout cDNA libraries BMC Genomics 2006 7 1 196 10.1186/1471-2164-7-196 16887034 PMC1564016

[B19] Salem M Rexroad C Wang J Thorgaard G Yao J Characterization of the rainbow trout transcriptome using Sanger and 454-pyrosequencing approaches BMC Genomics 2010 11 1 564 10.1186/1471-2164-11-564 20942956 PMC3091713

[B20] Salem M Xiao C Womack J Rexroad CE Yao J MicroRNA repertoire for functional genome research in rainbow trout (Oncorhynchus mykiss) Marine Biotech 2009 12 4 410 429 10.1007/s10126-009-9232-z 19816740

[B21] Rise ML von Schalburg KR Brown GD Mawer MA Devlin RH Kuipers N Busby M Beetz-Sargent M Alberto R Gibbs AR Development and Application of a Salmonid EST Database and cDNA Microarray: Data Mining and Interspecific Hybridization Characteristics Genome Res 2004 14 3 478 490 10.1101/gr.1687304 14962987 PMC353236

[B22] Salem M Kenney B Rexroad CE Yao J Development of a 37 k high-density oligonucleotide microarray: a new tool for functional genome research in rainbow trout Journal of Fish Biology 2008 72 2187 2206 10.1111/j.1095-8649.2008.01860.x

[B23] Phillips R Keatley K Morasch M Ventura A Lubieniecki K Koop B Danzmann R Davidson W Assignment of Atlantic salmon (Salmo salar) linkage groups to specific chromosomes: Conservation of large syntenic blocks corresponding to whole chromosome arms in rainbow trout (Oncorhynchus mykiss) BMC Genetics 2009 10 1 46 10.1186/1471-2156-10-46 19689812 PMC2734554

[B24] Phillips RB Nichols KM DeKoning JJ Morasch MR Keatley KA Rexroad C Gahr SA Danzmann RG Drew RE Thorgaard GH Assignment of rainbow trout linkage groups to specific chromosomes Genetics 2006 174 3 1661 1670 10.1534/genetics.105.055269 16951085 PMC1667062

[B25] Nichols KM Bartholomew J Thorgaard GH Mapping multiple genetic loci associated with Ceratomyxa shasta resistance in Oncorhynchus mykiss Dis Aquat Organ 2003 56 2 145 154 10.3354/dao056145 14598990

[B26] Barroso RM Wheeler PA LaPatra SE Drew RE Thorgaard GH QTL for IHNV resistance and growth identified in a rainbow (Oncorhynchus mykiss)xYellowstone cutthroat (Oncorhynchus Aquaculture 2008 277 3 156 163 10.1016/j.aquaculture.2008.03.001

[B27] Rodriguez MF LaPatra S Williams S Famula T May B Genetic markers associated with resistance to infectious hematopoietic necrosis in rainbow and steelhead trout Aquaculture 2004 241 1 93 115 10.1016/j.aquaculture.2004.08.003

[B28] Ozaki A Sakamoto T Khoo S Nakamura K Coimbra MR Akutsu T Okamoto N Quantitative trait loci (QTLs) associated with resistance/susceptibility to infectious pancreatic necrosis virus (IPNV) in rainbow trout (Oncorhynchus mykiss) Mol Genet Genomics 2001 265 1 23 31 10.1007/s004380000392 11370869

[B29] Baerwald MR Petersen JL Hedrick RP Schisler GJ May B A major effect quantitative trait locus for whirling disease resistance identified in rainbow trout (Oncorhynchus mykiss) Heredity 2010 in press 21048672 10.1038/hdy.2010.137 PMC3186244

[B30] Zimmerman A Evenhuis J Thorgaard G Ristow S A single major chromosomal region controls natural killer cell-like activity in rainbow trout Immunogenetics 2004 55 12 825 835 10.1007/s00251-004-0645-6 14968267

[B31] Perry GM Danzmann RG Ferguson MM Gibson JP Quantitative trait loci for upper thermal tolerance in outbred strains of rainbow trout (Oncorhynchus mykiss) Heredity 2001 86 Pt 3 333 341 10.1046/j.1365-2540.2001.00838.x 11488970

[B32] Perry GM Ferguson MM Sakamoto T Danzmann RG Sex-linked quantitative trait loci for thermotolerance and length in the rainbow trout J Hered 2005 96 2 97 107 10.1093/jhered/esi019 15653562

[B33] Nichols KM Broman KW Sundin K Young JM Wheeler PA Thorgaard GH Quantitative trait loci × maternal cytoplasmic environment interaction for development rate in Oncorhynchus mykiss Genetics 2007 175 1 335 347 10.1534/genetics.106.064311 17057232 PMC1774986

[B34] Sundin K Brown KH Drew RE Nichols KM Wheeler PA Thorgaard GH Genetic analysis of a development rate QTL in backcrosses of clonal rainbow trout, Oncorhynchus mykiss Aquaculture 2005 247 1-4 75 83 10.1016/j.aquaculture.2005.02.054

[B35] O'Malley KG Sakamoto T Danzmann RG Ferguson MM Quantitative trait Loci for spawning date and body weight in rainbow trout: testing for conserved effects across ancestrally duplicated chromosomes J Hered 2003 94 4 273 284 12920098 10.1093/jhered/esg067

[B36] Sakamoto T Danzmann RG Okamoto N Ferguson MM Ihssen PE Linkage analysis of quantitative trait loci associated with spawning time in rainbow trout (Oncorhynchus mykiss) Aquaculture 1999 173 1-4 33 43 10.1016/S0044-8486(98)00463-3

[B37] Drew RE Schwabl H Wheeler PA Thorgaard GH Detection of QTL influencing cortisol levels in rainbow trout (Oncorhynchus mykiss) Aquaculture 2007 272 Supplement 1 S183 S194 10.1016/j.aquaculture.2007.08.025

[B38] Haidle L Janssen J Gharbi K Moghadam H Ferguson M Danzmann R Determination of Quantitative Trait Loci (QTL) for Early Maturation in Rainbow Trout (Oncorhynchus mykiss) Marine Biotechnology 2008 10 5 579 592 10.1007/s10126-008-9098-5 18491191 PMC2516301

[B39] Nichols KM Edo AF Wheeler PA Thorgaard GH The Genetic Basis of Smoltification-Related Traits in Oncorhynchus mykiss Genetics 2008 179 3 1559 1575 10.1534/genetics.107.084251 18562654 PMC2475755

[B40] Johnson NA Vallejo RL Silverstein JT Welch TJ Wiens GD Hallerman EM Palti Y Suggestive Association of Major Histocompatibility IB Genetic Markers with Resistance to Bacterial Cold Water Disease in Rainbow Trout (Oncorhynchus mykiss) Mar Biotechnol 2008 10 4 429 437 10.1007/s10126-007-9080-7 18274824

[B41] Rexroad C Vallejo R Estimates of linkage disequilibrium and effective population size in rainbow trout BMC Genetics 2009 10 1 83 10.1186/1471-2156-10-83 20003428 PMC2800115

[B42] Castano-Sanchez C Smith T Wiedmann R Vallejo R Salem M Yao J Rexroad C Single nucleotide polymorphism discovery in rainbow trout by deep sequencing of a reduced representation library BMC Genomics 2009 10 1 559 10.1186/1471-2164-10-559 19939274 PMC2790473

[B43] Ning Z Cox AJ Mullikin JC SSAHA: A Fast Search Method for Large DNA Databases Genome Research 2001 11 10 1725 1729 10.1101/gr.194201 11591649 PMC311141

[B44] Voorrips RE MapChart: software for the graphical presentation of linkage maps and QTLs J Hered 2002 93 1 77 78 10.1093/jhered/93.1.77 12011185

[B45] Quiniou S Waldbieser G Duke M A first generation BAC-based physical map of the channel catfish genome BMC Genomics 2007 8 1 40 10.1186/1471-2164-8-40 17284319 PMC1800894

[B46] Nelson WM Bharti AK Butler E Wei F Fuks G Kim H Wing RA Messing J Soderlund C Whole-Genome Validation of High-Information-Content Fingerprinting Plant Physiol 2005 139 1 27 38 10.1104/pp.105.061978 16166258 PMC1203355

[B47] Nievergelt CM Smith DW Kohlenberg JB Schork NJ Large-Scale Integration of Human Genetic and Physical Maps Genome Research 2004 14 6 1199 1205 10.1101/gr.1475304 15140834 PMC419799

[B48] Danzmann RG Cairney M Davidson WS Ferguson MM Gharbi K Guyomard R Holm LE Leder E Okamoto N Ozaki A A comparative analysis of the rainbow trout genome with 2 other species of fish (Arctic charr and Atlantic salmon) within the tetraploid derivative Salmonidae family (subfamily: Salmoninae) Genome 2006 48 1037 1051 10.1139/g05-067 16391673

[B49] Ewing B Green P Base-calling of automated sequencer traces using phred. II. Error probabilities Genome Res 1998 8 3 186 194 9521922

[B50] Kim UJ Birren BW Slepak T Mancino V Boysen C Kang HL Simon MI Shizuya H Construction and characterization of a human bacterial artificial chromosome library Genomics 1996 34 2 213 218 10.1006/geno.1996.0268 8661051

[B51] Benson G Tandem repeats finder: a program to analyze DNA sequences Nucleic Acids Res 1999 27 2 573 580 10.1093/nar/27.2.573 9862982 PMC148217

[B52] Rozen S Skaletsky H Primer3 on the www for general users and for biologist programmers Methods Mol Biol 2000 132 365 386 10547847 10.1385/1-59259-192-2:365

[B53] Boutin-Ganache I Raposo M Raymond M Deschepper CF M13-tailed primers improve the readability and usability of microsatellite analyses performed with two different allele-sizing methods Biotechniques 2001 31 1 24 26 28 11464515

[B54] Coulibaly I Gahr SA Palti Y Yao J Rexroad CE Genomic structure and expression of uncoupling protein 2 genes in rainbow trout (Oncorhynchus mykiss) BMC Genomics 2006 7 203 203 10.1186/1471-2164-7-203 16899121 PMC1559616

[B55] Palti Y Gahr SA Purcell MK Hadidi S Rexroad Iii CE Wiens GD Identification, characterization and genetic mapping of TLR7, TLR8a1 and TLR8a2 genes in rainbow trout (Oncorhynchus mykiss) Developmental & Comparative Immunology 2010 34 2 219 233 10.1016/j.dci.2009.10.002 19825389

[B56] Van Tassell CP Smith TP Matukumalli LK Taylor JF Schnabel RD Lawley CT Haudenschild CD Moore SS Warren WC Sonstegard TS SNP discovery and allele frequency estimation by deep sequencing of reduced representation libraries Nat Methods 2008 5 3 247 252 10.1038/nmeth.1185 18297082

[B57] Wiedmann RT Smith TP Nonneman DJ SNP discovery in swine by reduced representation and high throughput pyrosequencing BMC Genet 2008 9 81 10.1186/1471-2156-9-81 19055830 PMC2612698

[B58] Ramos AM Crooijmans RPMA Affara NA Amaral AJ Archibald AL Beever JE Bendixen C Churcher C Clark R Dehais P Design of a High Density SNP Genotyping Assay in the Pig Using SNPs Identified and Characterized by Next Generation Sequencing Technology PLoS ONE 2009 4 8 e6524 10.1371/journal.pone.0006524 19654876 PMC2716536

[B59] Matise TC Perlin M Chakravarti A Automated construction of genetic linkage maps using an expert system (MultiMap): a human genome linkage map Nat Genet 1994 6 4 384 390 10.1038/ng0494-384 8054979

[B60] Lander ES Green P Construction of multilocus genetic linkage maps in humans Proc Natl Acad Sci USA 1987 84 8 2363 2367 10.1073/pnas.84.8.2363 3470801 PMC304651

[B61] Lathrop GM Lalouel JM Julier C Ott J Strategies for multilocus linkage analysis in humans Proc Natl Acad Sci USA 1984 81 11 3443 3446 10.1073/pnas.81.11.3443 6587361 PMC345524

[B62] O'Connell JR Weeks DE PedCheck: a program for identification of genotype incompatibilities in linkage analysis Am J Hum Genet 1998 63 1 259 266 9634505 10.1086/301904 PMC1377228

[B63] O'Connell JR Weeks DE The VITESSE algorithm for rapid exact multilocus linkage analysis via genotype set-recoding and fuzzy inheritance Nat Genet 1995 11 4 402 408 7493020 10.1038/ng1295-402

