# Supplementary material for: A first generation integrated map of the rainbow trout genome
Source: BMC Genomics. 2011 Apr 7;12:180. doi: 10.1186/1471-2164-12-180 (PMC3079668; doi:10.1186/1471-2164-12-180)
Supplement: Additional file 2 — Table S1 [file 1471-2164-12-180-S2.DOC]

**Supplementary Tables – Immune Response Genes**

**Table S1.** PCR primers from immune gene sequences used in the BAC library screening to identify BACs and contigs that harbor the genes.

| **Gene/Locus** | **Accession** | **Forward Primer** | **Reverse Primer** |
| --- | --- | --- | --- |
| RtGST-1^a^ | EF445945 | TTCGAGGTTCTCCTCACTCTT | AAAGTTGCAACGTCGTCAG |
| TNF-New1^b^ | DQ218472 | ACTGAGTGAAGCCGTGTAT | TCTGTTGGCTCAATATGGG |
| IRAK4 | BX884289 | AGGAGATGTTTGATCGCTGTG | CCTCATTTGCCATGTATGCTG |
| CXCg | CA356423 | CGCTGAGCAACTATCGTCATT | CAAGCGTGCATGCACTAAATC |
| CXCf | BX085600 | CTAAAGAGGTTGGAAGCGTCACTC | CAGTGCAATATCCCAATCGG |
| CXCe | BX859166 | TGAGGCCATCTCTAATGAG | TAGAACAAGAAGCGTCTGC |
| CXCh | CX035482 | CTCGCTACTTATATTCACGATGGC | TTTCAGTAGTGTCGCAGTAATGGG |
| Light-V2B^b^ | DQ218470 | CCCAACTTCAAGACCAAACCCTAT | CAAGCTTCCCATCTTTGTACTCCA |
| Light-V2C^b^ | DQ218470 | TCCAGAAGGAAGGCTACTACTACG | GGGGTGATATCTCCTGGACTTA |
| NOD-1 | CA372055 | TGAACAGGCCAGCGTAGGAA | CAGCATCCACAGGAAGTTGGG |
| NOD-2 | CX244678 | CGGTTTTCTGCTGGATTGTGTC | AACTCTGGAGGTGTTGGATCTGG |
| LAA | DQ789362 | GTGCGTCCTAGAGTAAGACTC | GAACCCAACTGACATCCAGC |

^a^ Qiu GF, Weber GM, Rexroad CE, 3rd, Yao J: **Identification of RtGST-1, a novel germ cell-specific mRNA-like transcript predominantly expressed in early previtellogenic oocytes in rainbow trout (Oncorhynchus mykiss).** *Mol Reprod Dev* 2008, **75:**723-730.

^b^ Glenney GW, Wiens GD: **Early Diversification of the TNF Superfamily in Teleosts: Genomic Characterization and Expression Analysis.** *J Immunol* 2007, **178:**7955-7973.

**Table S2.** Microsatellite markers isolated from BACs that harbor immune response genes and three TLR9 SNPs.

| **Gene** | **Marker** | **Accession** | **Forward Primer** | **Reverse Primer** |
| --- | --- | --- | --- | --- |
| TLR1a [1] | OMM3154 | GF101807 | ATGCGTCCCAGTCCAAT | GGATGTGTTGCCTAACCAC |
| TLR1a [1] | OMM3155 | GF101808 | TGCGTGTCATGCGTGTATGTGTAT | GGCAATGATGGAGATGGTAATGTG |
| TLR1b (ψ) [1] | OMM3172 | GF101810 | TAGCAAAGGCAGAGAAACGTG | TCATGCAGCGTCCTTATTCAC |
| TLR1c (ψ) [1] | OMM3168 | GF101809 | GGGTTATAAGACACCTACCTC | AGTCTCTTATCTCCCACACTC |
| TLR8a2 [2] | OMM3183 | GF100699 | CATCACGTCTCGCTCAAAG | TTTCCGACACGTCTTGG |
| TLR8a1 & TLR7 [2] | OMM3184 | GF100700 | GGTATACCGCCCAAGCTTAAC | GGCGGAGTTTCAGGGGTATT |
| LDA [3] | OMM3158 | AB258536 | TAGCCAACCAGGGACTTTC | GTGTTGACAGTCTGCGAAGAG |
| TLR9 [4, 5] | c.1233C>T | EU627195 | CATTGCCATACGCACCGGTA | TGGATGATGCCAGGGCTCAT |
| TLR9 [4, 5] | c.1489C>T | EU627195 | TAAGAGCTTTCAGAACCAGAACCG | ACTTAACGCCTCCAGATTGGGGA |
| TLR9 [4, 5] | c.1992C>T | EU627195 | AAGAACCTCCCCAATCTGG | GATGTTCTCCCAGGGGAAA |

1. Palti Y, Rodriguez MF, Gahr SA, Purcell MK, Rexroad Iii CE, Wiens GD: **Identification, characterization and genetic mapping of TLR1 loci in rainbow trout (Oncorhynchus mykiss).** *Fish & Shellfish Immunology* 2010, **28:**918-926.

2. Palti Y, Gahr SA, Purcell MK, Hadidi S, Rexroad Iii CE, Wiens GD: **Identification, characterization and genetic mapping of TLR7, TLR8a1 and TLR8a2 genes in rainbow trout (Oncorhynchus mykiss).** *Developmental & Comparative Immunology* 2010, **34:**219-233.

3. Dijkstra J, Katagiri T, Hosomichi K, Yanagiya K, Inoko H, Ototake M, Aoki T, Hashimoto K, Shiina T: **A third broad lineage of major histocompatibility complex (MHC) class I in teleost fish; MHC class II linkage and processed genes.** *Immunogenetics* 2007, **59:**305-321.

4. Ortega-Villaizan M, Chico V, Falco A, Perez L, Coll JM, Estepa A: **The rainbow trout TLR9 gene and its role in the immune responses elicited by a plasmid encoding the glycoprotein G of the viral haemorrhagic septicaemia rhabdovirus (VHSV).** *Molecular Immunology* 2009, **46:**1710-1717.

5. Kongchum P, C. E. Rexroad I, Hallerman EM, David L, Palti Y: **Single nucleotide polymorphism identification, genetic mapping and tissue expression of the rainbow trout TLR9 gene.** *Animal Genetics* 2009, **40:**1001.
