# Supplementary material for: A first generation integrated map of the rainbow trout genome
Source: BMC Genomics. 2011 Apr 7;12:180. doi: 10.1186/1471-2164-12-180 (PMC3079668; doi:10.1186/1471-2164-12-180)
Supplement: Additional file 4 — chromosome maps [file 1471-2164-12-180-S4.PDF]

# Omy1

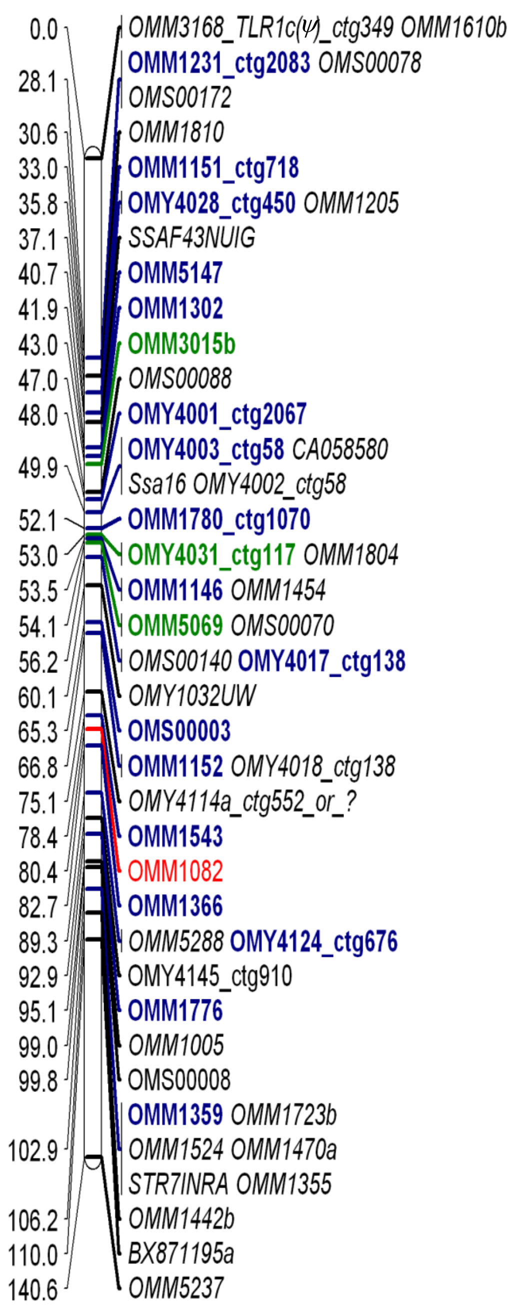
0.0
28.1
30.6
33.0
35.8
37.1
40.7
41.9
43.0
47.0
48.0
49.9
52.1
53.0
53.5
54.1
56.2
60.1
65.3
66.8
75.1
78.4
80.4
82.7
89.3
92.9
95.1
99.0
99.8
102.9
106.2
110.0
140.6
OMM3168_TLR1c(ψ)_ctg349 OMM1610b
OMM1231_ctg2083 OMS00078
OMS00172
OMM1810
OMM1151_ctg718
OMY4028_ctg450 OMM1205
SSAF43NUIG
OMM5147
OMM1302
OMM3015b
OMS00088
OMY4001_ctg2067
OMY4003_ctg58 CA058580
Ssa16 OMY4002_ctg58
OMM1780_ctg1070
OMY4031_ctg117 OMM1804
OMM1146 OMM1454
OMM5069 OMS00070
OMS00140 OMY4017_ctg138
OMY1032UW
OMS00003
OMM1152 OMY4018_ctg138
OMY4114a_ctg552_or_?
OMM1543
OMM1082
OMM1366
OMM5288 OMY4124_ctg676
OMY4145_ctg910
OMM1776
OMM1005
OMS00008
OMM1359 OMM1723b
OMM1524 OMM1470a
STR7INRA OMM1355
OMM1442b
BX871195a
OMM5237

## Omy2

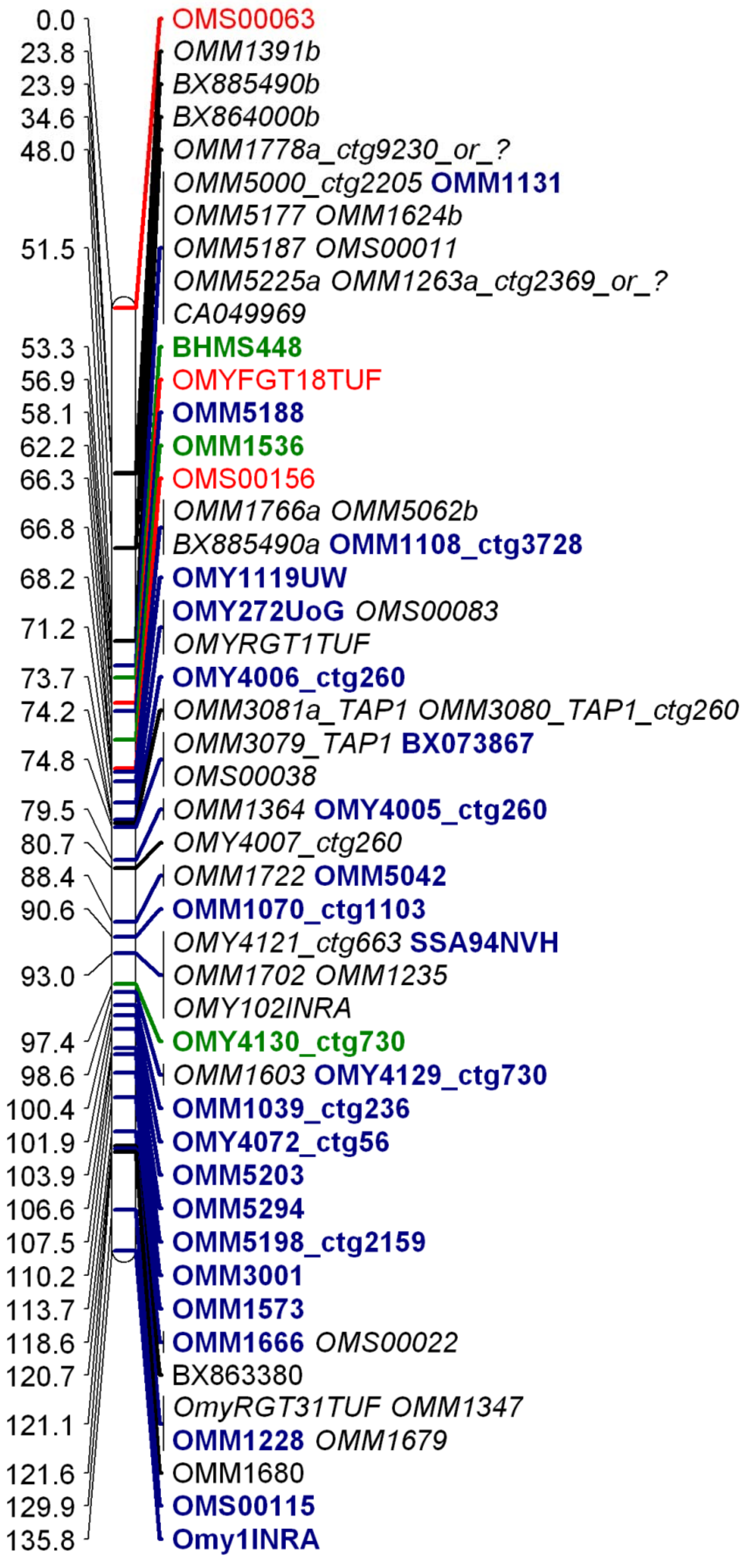

0.0
23.8
23.9
34.6
48.0
51.5
53.3
56.9
58.1
62.2
66.3
66.8
68.2
71.2
73.7
74.2
74.8
79.5
80.7
88.4
90.6
93.0
97.4
98.6
100.4
101.9
103.9
106.6
107.5
110.2
113.7
118.6
120.7
121.1
121.6
129.9
135.8
OMS00063
OMM1391b
BX885490b
BX864000b
OMM1778a_ctg9230_or_?
OMM5000_ctg2205 OMM1131
OMM5177 OMM1624b
OMM5187 OMS00011
OMM5225a OMM1263a_ctg2369_or_?
CA049969
BHMS448
OMYFGT18TUF
OMM5188
OMM1536
OMS00156
OMM1766a OMM5062b
BX885490a OMM1108_ctg3728
OMY1119UW
OMY272UoG OMS00083
OMYRGT1TUF
OMY4006_ctg260
OMM3081a_TAP1 OMM3080_TAP1_ctg260
OMM3079_TAP1 BX073867
OMS00038
OMM1364 OMY4005_ctg260
OMY4007_ctg260
OMM1722 OMM5042
OMM1070_ctg1103
OMY4121_ctg663 SSA94NVH
OMM1702 OMM1235
OMY102INRA
OMY4130_ctg730
OMM1603 OMY4129_ctg730
OMM1039_ctg236
OMY4072_ctg56
OMM5203
OMM5294
OMM5198_ctg2159
OMM3001
OMM1573
OMM1666 OMS00022
BX863380
OmyRGT31TUF OMM1347
OMM1228 OMM1679
OMM1680
OMS00115
Omy1INRA

## Omy3

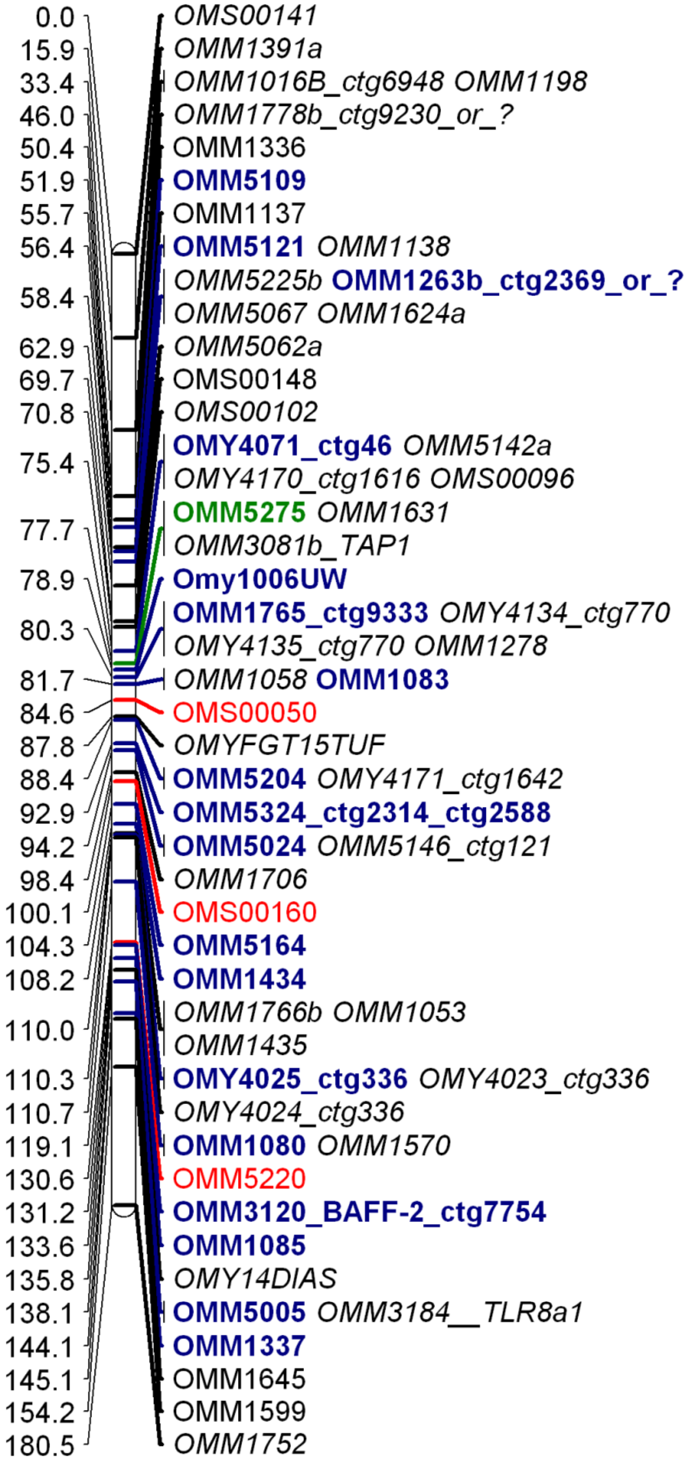

0.0 OMS00141
15.9 OMM1391a
33.4 OMM1016B_ctg6948 OMM1198
46.0 OMM1778b_ctg9230_or_?
50.4 OMM1336
51.9 OMM5109
55.7 OMM1137
56.4 OMM5121 OMM1138
58.4 OMM5225b OMM1263b_ctg2369_or_?
OMM5067 OMM1624a
62.9 OMM5062a
69.7 OMS00148
70.8 OMS00102
75.4 OMY4071_ctg46 OMM5142a
OMY4170_ctg1616 OMS00096
77.7 OMM5275 OMM1631
OMM3081b_TAP1
78.9 Omy1006UW
80.3 OMM1765_ctg9333 OMY4134_ctg770
OMY4135_ctg770 OMM1278
81.7 OMM1058 OMM1083
84.6 OMS00050
87.8 OMYFGT15TUF
88.4 OMM5204 OMY4171_ctg1642
92.9 OMM5324_ctg2314_ctg2588
94.2 OMM5024 OMM5146_ctg121
98.4 OMM1706
100.1 OMS00160
104.3 OMM5164
108.2 OMM1434
110.0 OMM1766b OMM1053
OMM1435
110.3 OMY4025_ctg336 OMY4023_ctg336
110.7 OMY4024_ctg336
119.1 OMM1080 OMM1570
130.6 OMM5220
131.2 OMM3120_BAFF-2_ctg7754
133.6 OMM1085
135.8 OMY14DIAS
138.1 OMM5005 OMM3184__TLR8a1
144.1 OMM1337
145.1 OMM1645
154.2 OMM1599
180.5 OMM1752

# Omy4

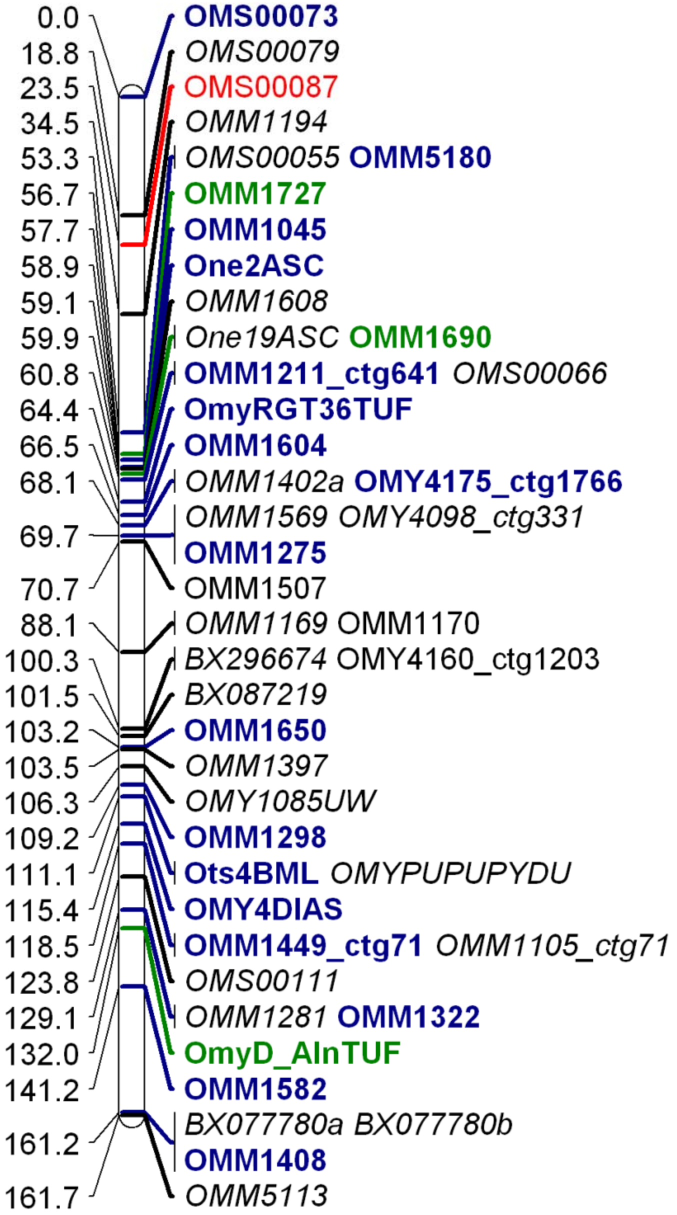
0.0
18.8
23.5
34.5
53.3
56.7
57.7
58.9
59.1
59.9
60.8
64.4
66.5
68.1
69.7
70.7
88.1
100.3
101.5
103.2
103.5
106.3
109.2
111.1
115.4
118.5
123.8
129.1
132.0
141.2
161.2
161.7
OMS00073
OMS00079
OMS00087
OMM1194
OMS00055 OMM5180
OMM1727
OMM1045
One2ASC
OMM1608
One19ASC OMM1690
OMM1211_ctg641 OMS00066
OmyRGT36TUF
OMM1604
OMM1402a OMY4175_ctg1766
OMM1569 OMY4098_ctg331
OMM1275
OMM1507
OMM1169 OMM1170
BX296674 OMY4160_ctg1203
BX087219
OMM1650
OMM1397
OMY1085UW
OMM1298
Ots4BML OMYPUPUPYDU
OMY4DIAS
OMM1449_ctg71 OMM1105_ctg71
OMS00111
OMM1281 OMM1322
OmyD_AlnTUF
OMM1582
BX077780a BX077780b
OMM1408
OMM5113

## Omy5

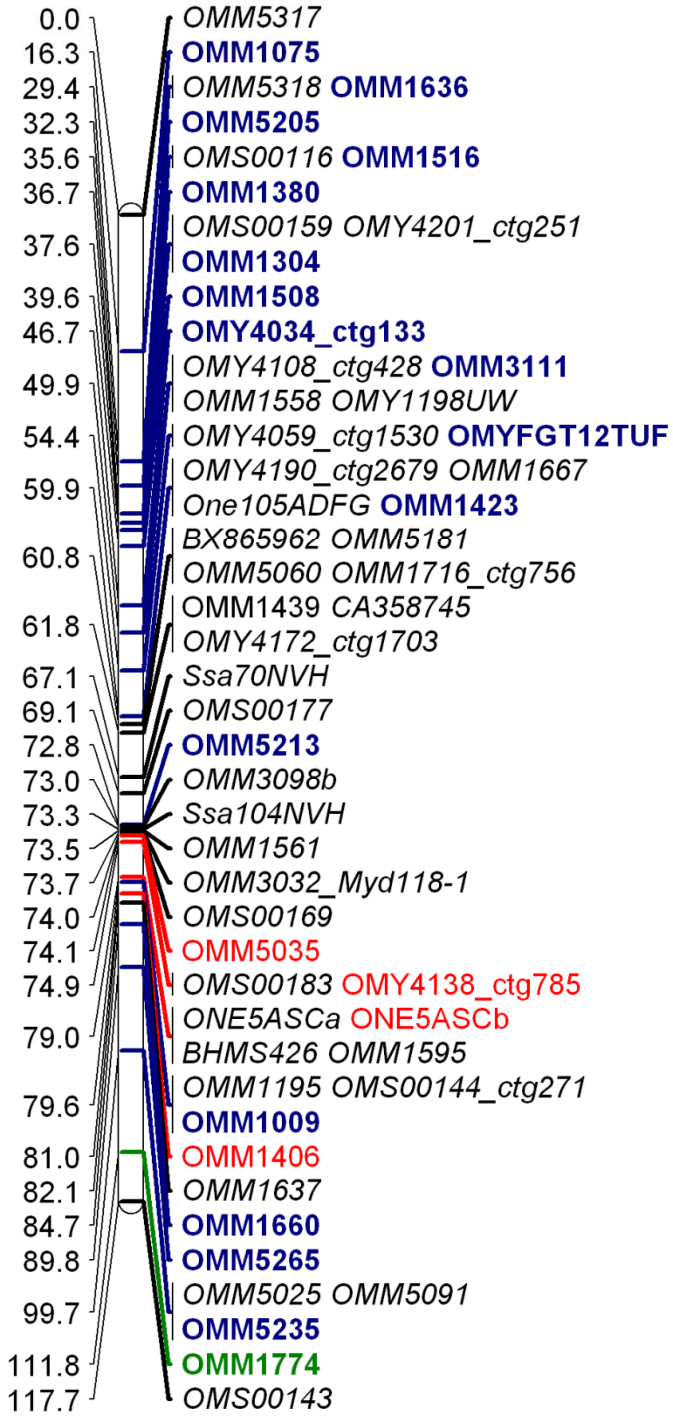
0.0 OMM5317
16.3 OMM1075
29.4 OMM5318 OMM1636
32.3 OMM5205
35.6 OMS00116 OMM1516
36.7 OMM1380
37.6 OMS00159 OMY4201_ctg251
OMM1304
39.6 OMM1508
46.7 OMY4034_ctg133
49.9 OMY4108_ctg428 OMM3111
OMM1558 OMY1198UW
54.4 OMY4059_ctg1530 OMYFGT12TUF
59.9 OMY4190_ctg2679 OMM1667
One105ADFG OMM1423
60.8 BX865962 OMM5181
OMM5060 OMM1716_ctg756
61.8 OMM1439 CA358745
OMY4172_ctg1703
67.1 Ssa70NVH
69.1 OMS00177
72.8 OMM5213
73.0 OMM3098b
73.3 Ssa104NVH
73.5 OMM1561
73.7 OMM3032_Myd118-1
74.0 OMS00169
74.1 OMM5035
74.9 OMS00183 OMY4138_ctg785
79.0 ONE5ASCa ONE5ASCb
BHMS426 OMM1595
79.6 OMM1195 OMS00144_ctg271
OMM1009
81.0 OMM1406
82.1 OMM1637
84.7 OMM1660
89.8 OMM5265
99.7 OMM5025 OMM5091
OMM5235
111.8 OMM1774
117.7 OMS00143

## Omy6

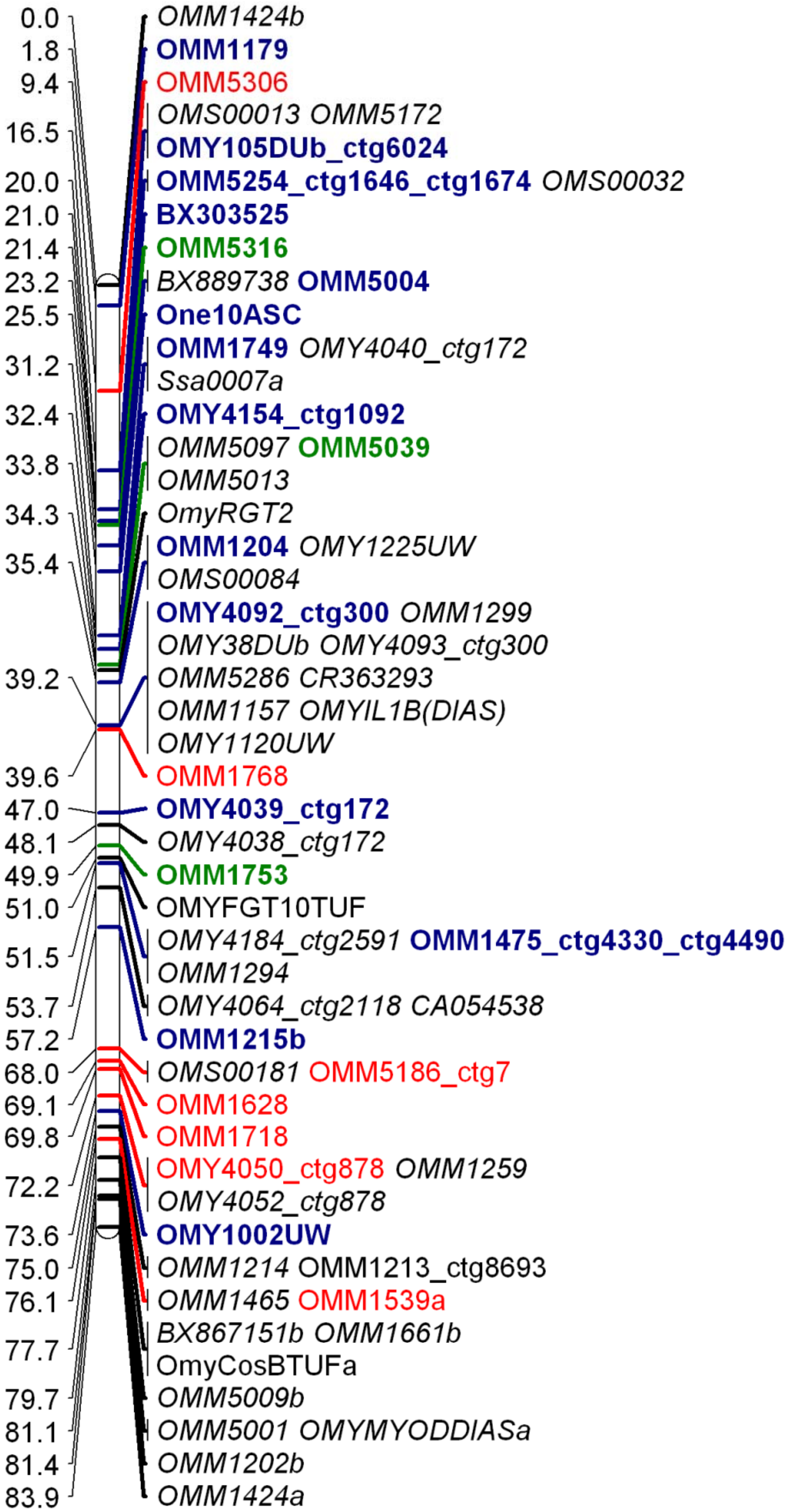
0.0
1.8
9.4
16.5
20.0
21.0
21.4
23.2
25.5
31.2
32.4
33.8
34.3
35.4
39.2
39.6
47.0
48.1
49.9
51.0
51.5
53.7
57.2
68.0
69.1
69.8
72.2
73.6
75.0
76.1
77.7
79.7
81.1
81.4
83.9
OMM1424b
OMM1179
OMM5306
OMS00013 OMM5172
OMY105DUb_ctg6024
OMM5254_ctg1646_ctg1674 OMS00032
BX303525
OMM5316
BX889738 OMM5004
One10ASC
OMM1749 OMY4040_ctg172
Ssa0007a
OMY4154_ctg1092
OMM5097 OMM5039
OMM5013
OmyRGT2
OMM1204 OMY1225UW
OMS00084
OMY4092_ctg300 OMM1299
OMY38DUb OMY4093_ctg300
OMM5286 CR363293
OMM1157 OMYIL1B(DIAS)
OMY1120UW
OMM1768
OMY4039_ctg172
OMY4038_ctg172
OMM1753
OMYFGT10TUF
OMY4184_ctg2591 OMM1475_ctg4330_ctg4490
OMM1294
OMY4064_ctg2118 CA054538
OMM1215b
OMS00181 OMM5186_ctg7
OMM1628
OMM1718
OMY4050_ctg878 OMM1259
OMY4052_ctg878
OMY1002UW
OMM1214 OMM1213_ctg8693
OMM1465 OMM1539a
BX867151b OMM1661b
OmyCosBTUFa
OMM5009b
OMM5001 OMYMYODDIASa
OMM1202b
OMM1424a

## Omy7

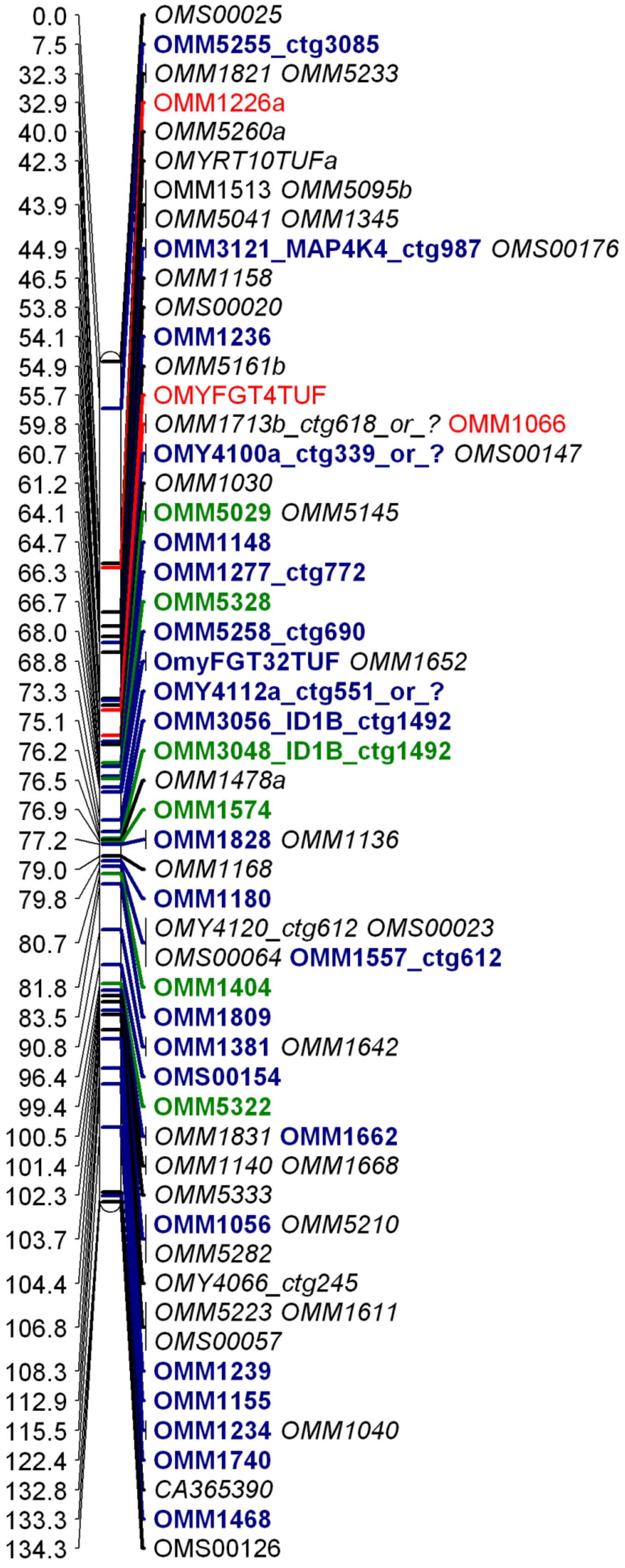
0.0 OMS00025
7.5 OMM5255_ctg3085
32.3 OMM1821 OMM5233
32.9 OMM1226a
40.0 OMM5260a
42.3 OMYRT10TUFa
43.9 OMM1513 OMM5095b
OMM5041 OMM1345
44.9 OMM3121_MAP4K4_ctg987 OMS00176
46.5 OMM1158
53.8 OMS00020
54.1 OMM1236
54.9 OMM5161b
55.7 OMYFGT4TUF
59.8 OMM1713b_ctg618_or_? OMM1066
60.7 OMY4100a_ctg339_or_? OMS00147
61.2 OMM1030
64.1 OMM5029 OMM5145
64.7 OMM1148
66.3 OMM1277_ctg772
66.7 OMM5328
68.0 OMM5258_ctg690
68.8 OmyFGT32TUF OMM1652
73.3 OMY4112a_ctg551_or_?
75.1 OMM3056_ID1B_ctg1492
76.2 OMM3048_ID1B_ctg1492
76.5 OMM1478a
76.9 OMM1574
77.2 OMM1828 OMM1136
79.0 OMM1168
79.8 OMM1180
80.7 OMY4120_ctg612 OMS00023
OMS00064 OMM1557_ctg612
81.8 OMM1404
83.5 OMM1809
90.8 OMM1381 OMM1642
96.4 OMS00154
99.4 OMM5322
100.5 OMM1831 OMM1662
101.4 OMM1140 OMM1668
102.3 OMM5333
103.7 OMM1056 OMM5210
OMM5282
104.4 OMY4066_ctg245
106.8 OMM5223 OMM1611
OMS00057
108.3 OMM1239
112.9 OMM1155
115.5 OMM1234 OMM1040
122.4 OMM1740
132.8 CA365390
133.3 OMM1468
134.3 OMS00126

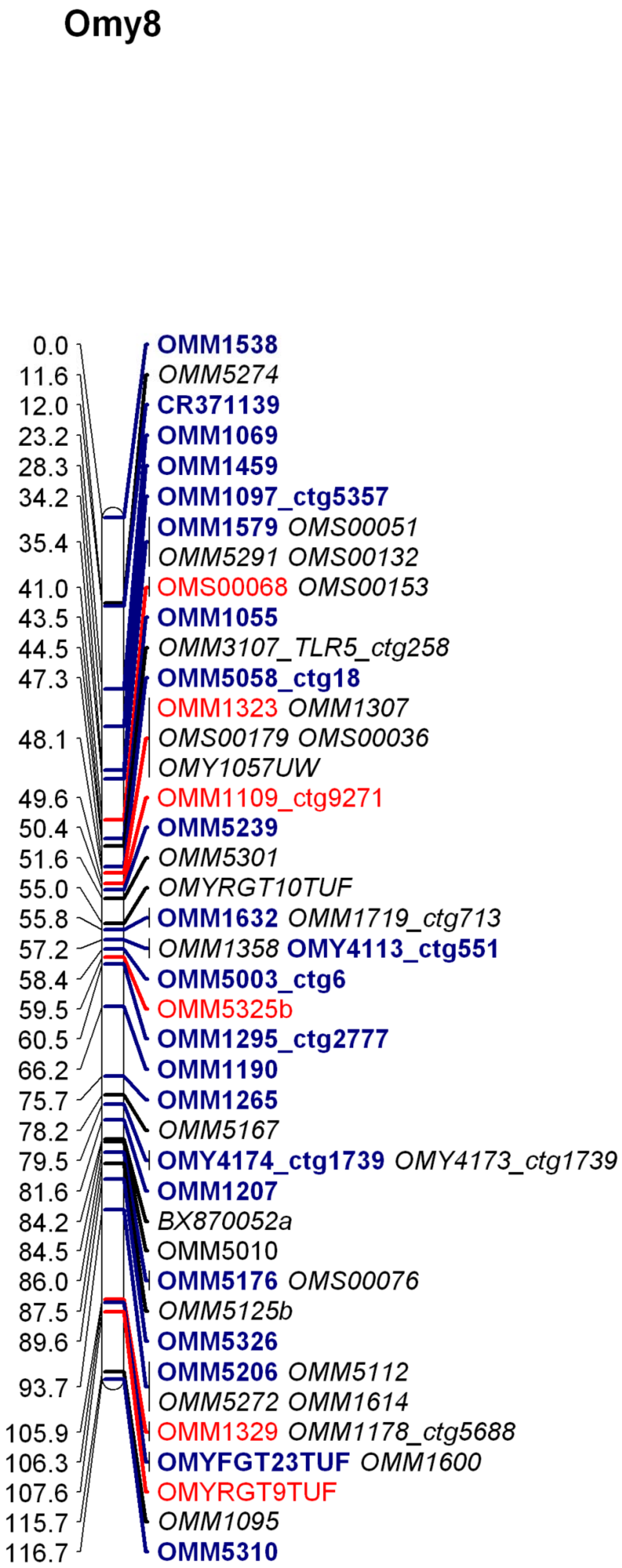
Omy8
0.0
11.6
12.0
23.2
28.3
34.2
35.4
41.0
43.5
44.5
47.3
48.1
49.6
50.4
51.6
55.0
55.8
57.2
58.4
59.5
60.5
66.2
75.7
78.2
79.5
81.6
84.2
84.5
86.0
87.5
89.6
93.7
105.9
106.3
107.6
115.7
116.7
OMM1538
OMM5274
CR371139
OMM1069
OMM1459
OMM1097_ctg5357
OMM1579 OMS00051
OMM5291 OMS00132
OMS00068 OMS00153
OMM1055
OMM3107_TLR5_ctg258
OMM5058_ctg18
OMM1323 OMM1307
OMS00179 OMS00036
OMY1057UW
OMM1109_ctg9271
OMM5239
OMM5301
OMYRGT10TUF
OMM1632 OMM1719_ctg713
OMM1358 OMY4113_ctg551
OMM5003_ctg6
OMM5325b
OMM1295_ctg2777
OMM1190
OMM1265
OMM5167
OMY4174_ctg1739 OMY4173_ctg1739
OMM1207
BX870052a
OMM5010
OMM5176 OMS00076
OMM5125b
OMM5326
OMM5206 OMM5112
OMM5272 OMM1614
OMM1329 OMM1178_ctg5688
OMYFGT23TUF OMM1600
OMYRGT9TUF
OMM1095
OMM5310

## Omy9

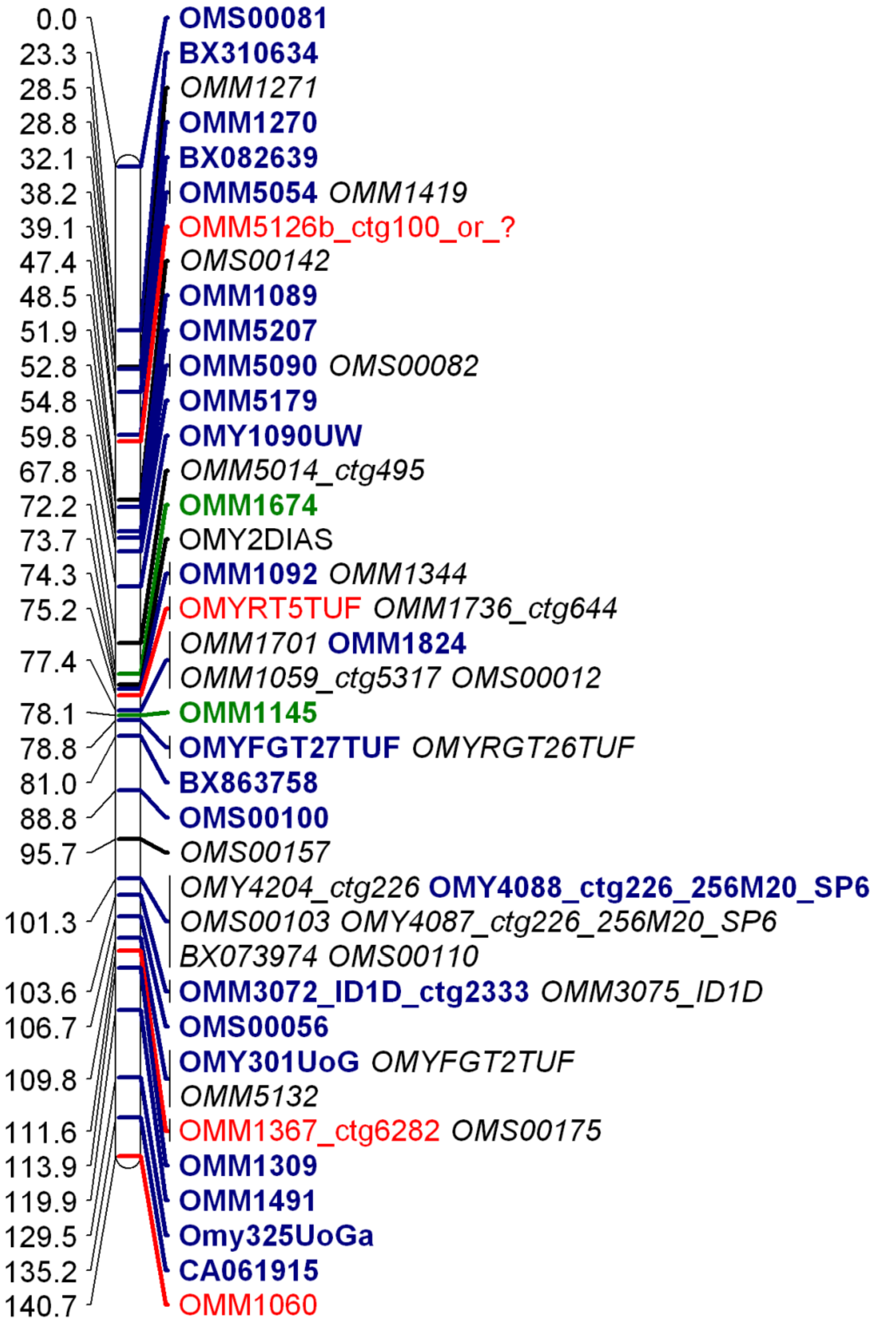

0.0 OMS00081
23.3 BX310634
28.5 OMM1271
28.8 OMM1270
32.1 BX082639
38.2 OMM5054 OMM1419
39.1 OMM5126b_ctg100_or_?
47.4 OMS00142
48.5 OMM1089
51.9 OMM5207
52.8 OMM5090 OMS00082
54.8 OMM5179
59.8 OMY1090UW
67.8 OMM5014_ctg495
72.2 OMM1674
73.7 OMY2DIAS
74.3 OMM1092 OMM1344
75.2 OMYRT5TUF OMM1736_ctg644
77.4 OMM1701 OMM1824
OMM1059_ctg5317 OMS00012
78.1 OMM1145
78.8 OMYFGT27TUF OMYRGT26TUF
81.0 BX863758
88.8 OMS00100
95.7 OMS00157
101.3 OMY4204_ctg226 OMY4088_ctg226_256M20_SP6
OMS00103 OMY4087_ctg226_256M20_SP6
BX073974 OMS00110
103.6 OMM3072_ID1D_ctg2333 OMM3075_ID1D
106.7 OMS00056
109.8 OMY301UoG OMYFGT2TUF
OMM5132
111.6 OMM1367_ctg6282 OMS00175
113.9 OMM1309
119.9 OMM1491
129.5 Omy325UoGa
135.2 CA061915
140.7 OMM1060

## Omy10

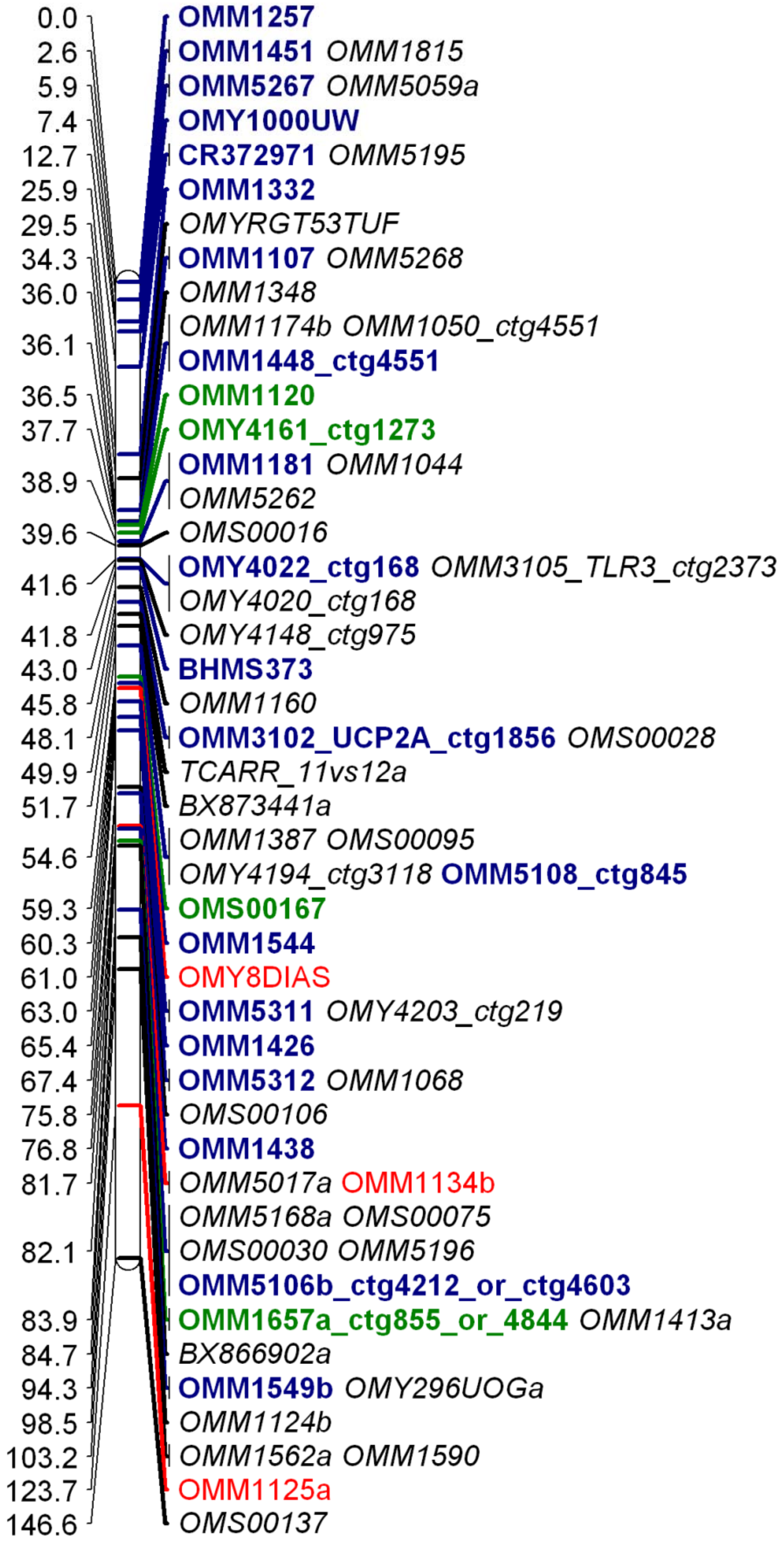

0.0 OMM1257
2.6 OMM1451 OMM1815
5.9 OMM5267 OMM5059a
7.4 OMY1000UW
12.7 CR372971 OMM5195
25.9 OMM1332
29.5 OMYRGT53TUF
34.3 OMM1107 OMM5268
36.0 OMM1348
OMM1174b OMM1050_ctg4551
36.1 OMM1448_ctg4551
36.5 OMM1120
37.7 OMY4161_ctg1273
OMM1181 OMM1044
38.9 OMM5262
39.6 OMS00016
OMY4022_ctg168 OMM3105_TLR3_ctg2373
41.6 OMY4020_ctg168
41.8 OMY4148_ctg975
43.0 BHMS373
45.8 OMM1160
48.1 OMM3102_UCP2A_ctg1856 OMS00028
49.9 TCARR_11vs12a
51.7 BX873441a
OMM1387 OMS00095
54.6 OMY4194_ctg3118 OMM5108_ctg845
59.3 OMS00167
60.3 OMM1544
61.0 OMY8DIAS
63.0 OMM5311 OMY4203_ctg219
65.4 OMM1426
67.4 OMM5312 OMM1068
75.8 OMS00106
76.8 OMM1438
81.7 OMM5017a OMM1134b
OMM5168a OMS00075
82.1 OMS00030 OMM5196
OMM5106b_ctg4212_or_ctg4603
83.9 OMM1657a_ctg855_or_4844 OMM1413a
84.7 BX866902a
94.3 OMM1549b OMY296UOGa
98.5 OMM1124b
103.2 OMM1562a OMM1590
123.7 OMM1125a
146.6 OMS00137

# Omy11

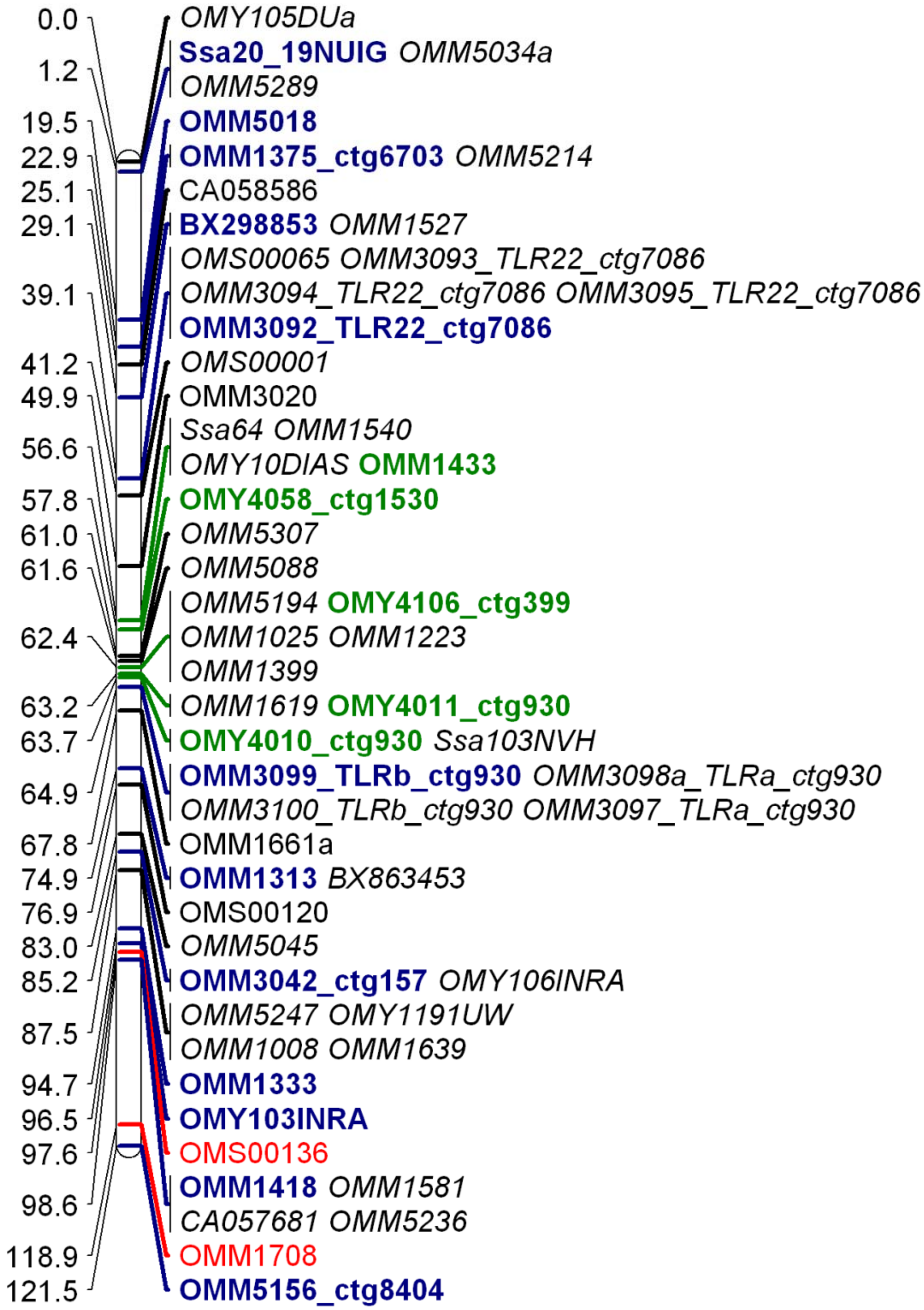

0.0
1.2
19.5
22.9
25.1
29.1
39.1
41.2
49.9
56.6
57.8
61.0
61.6
62.4
63.2
63.7
64.9
67.8
74.9
76.9
83.0
85.2
87.5
94.7
96.5
97.6
98.6
118.9
121.5
OMY105DUa
Ssa20_19NUIG OMM5034a
OMM5289
OMM5018
OMM1375_ctg6703 OMM5214
CA058586
BX298853 OMM1527
OMS00065 OMM3093_TLR22_ctg7086
OMM3094_TLR22_ctg7086 OMM3095_TLR22_ctg7086
OMM3092_TLR22_ctg7086
OMS00001
OMM3020
Ssa64 OMM1540
OMY10DIAS OMM1433
OMY4058_ctg1530
OMM5307
OMM5088
OMM5194 OMY4106_ctg399
OMM1025 OMM1223
OMM1399
OMM1619 OMY4011_ctg930
OMY4010_ctg930 Ssa103NVH
OMM3099_TLRb_ctg930 OMM3098a_TLRa_ctg930
OMM3100_TLRb_ctg930 OMM3097_TLRa_ctg930
OMM1661a
OMM1313 BX863453
OMS00120
OMM5045
OMM3042_ctg157 OMY106INRA
OMM5247 OMY1191UW
OMM1008 OMM1639
OMM1333
OMY103INRA
OMS00136
OMM1418 OMM1581
CA057681 OMM5236
OMM1708
OMM5156_ctg8404

## Omy12

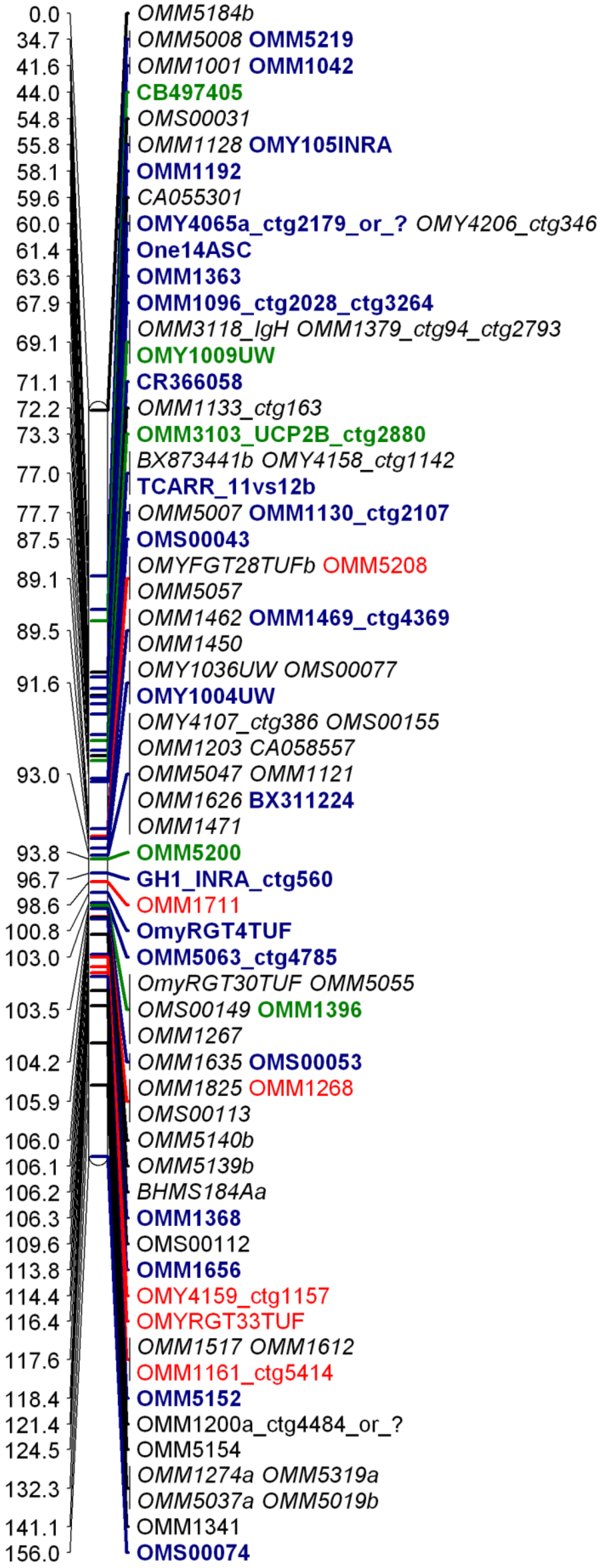
0.0 OMM5184b
34.7 OMM5008 OMM5219
41.6 OMM1001 OMM1042
44.0 CB497405
54.8 OMS00031
55.8 OMM1128 OMY105INRA
58.1 OMM1192
59.6 CA055301
60.0 OMY4065a_ctg2179_or_? OMY4206_ctg346
61.4 One14ASC
63.6 OMM1363
67.9 OMM1096_ctg2028_ctg3264
69.1 OMM3118_IgH OMM1379_ctg94_ctg2793
OMY1009UW
71.1 CR366058
72.2 OMM1133_ctg163
73.3 OMM3103_UCP2B_ctg2880
77.0 BX873441b OMY4158_ctg1142
TCARR_11vs12b
77.7 OMM5007 OMM1130_ctg2107
87.5 OMS00043
89.1 OMYFGT28TUFb OMM5208
OMM5057
89.5 OMM1462 OMM1469_ctg4369
OMM1450
91.6 OMY1036UW OMS00077
OMY1004UW
OMY4107_ctg386 OMS00155
OMM1203 CA058557
93.0 OMM5047 OMM1121
OMM1626 BX311224
OMM1471
93.8 OMM5200
96.7 GH1_INRA_ctg560
98.6 OMM1711
100.8 OmyRGT4TUF
103.0 OMM5063_ctg4785
OmyRGT30TUF OMM5055
103.5 OMS00149 OMM1396
OMM1267
104.2 OMM1635 OMS00053
OMM1825 OMM1268
105.9 OMS00113
106.0 OMM5140b
106.1 OMM5139b
106.2 BHMS184Aa
106.3 OMM1368
109.6 OMS00112
113.8 OMM1656
114.4 OMY4159_ctg1157
116.4 OMYRGT33TUF
117.6 OMM1517 OMM1612
OMM1161_ctg5414
118.4 OMM5152
121.4 OMM1200a_ctg4484_or_?
124.5 OMM5154
132.3 OMM1274a OMM5319a
OMM5037a OMM5019b
141.1 OMM1341
156.0 OMS00074

## Omy13

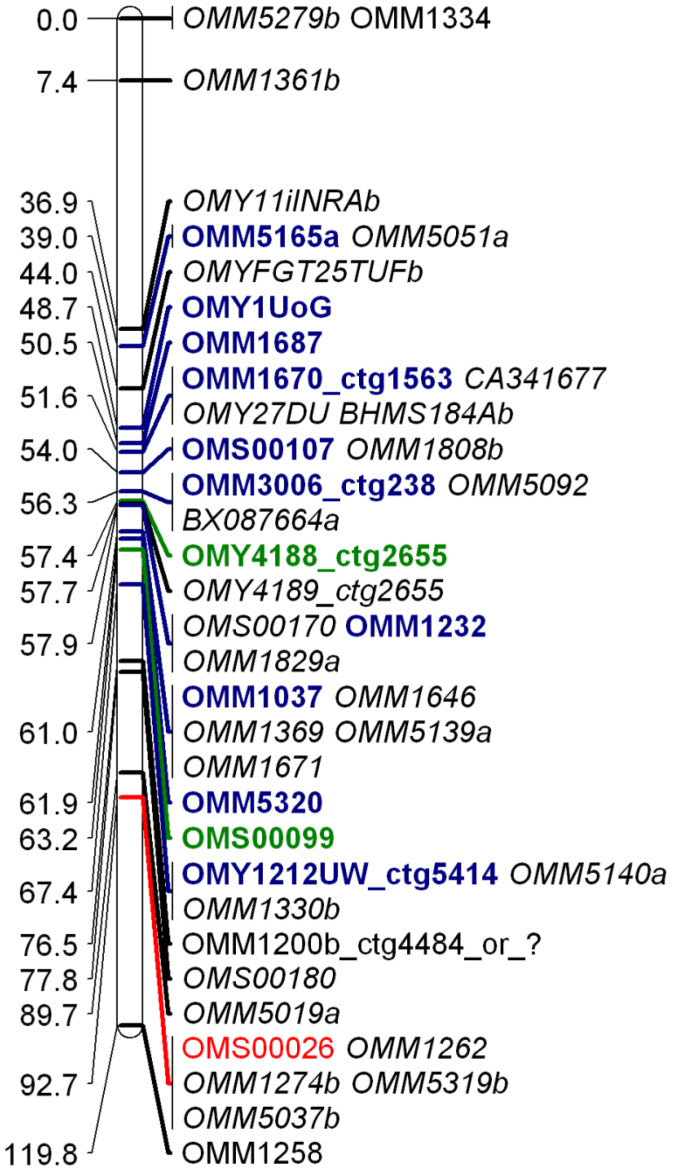
0.0
7.4
36.9
39.0
44.0
48.7
50.5
51.6
54.0
56.3
57.4
57.7
57.9
61.0
61.9
63.2
67.4
76.5
77.8
89.7
92.7
119.8
OMM5279b OMM1334
OMM1361b
OMY11iINRAb
OMM5165a OMM5051a
OMYFGT25TUFb
OMY1UoG
OMM1687
OMM1670_ctg1563 CA341677
OMY27DU BHMS184Ab
OMS00107 OMM1808b
OMM3006_ctg238 OMM5092
BX087664a
OMY4188_ctg2655
OMY4189_ctg2655
OMS00170 OMM1232
OMM1829a
OMM1037 OMM1646
OMM1369 OMM5139a
OMM1671
OMM5320
OMS00099
OMY1212UW_ctg5414 OMM5140a
OMM1330b
OMM1200b_ctg4484_or_?
OMS00180
OMM5019a
OMS00026 OMM1262
OMM1274b OMM5319b
OMM5037b
OMM1258

## Omy14

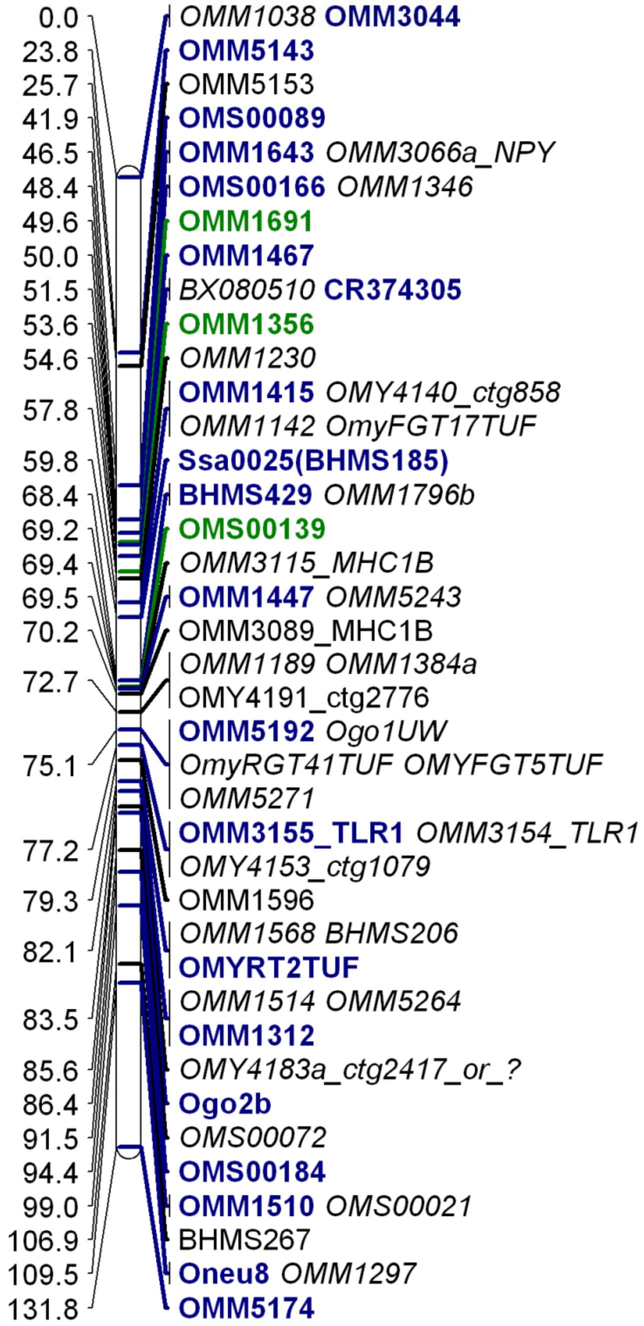

0.0 OMM1038 OMM3044
23.8 OMM5143
25.7 OMM5153
41.9 OMS00089
46.5 OMM1643 OMM3066a_NPY
48.4 OMS00166 OMM1346
49.6 OMM1691
50.0 OMM1467
51.5 BX080510 CR374305
53.6 OMM1356
54.6 OMM1230
57.8 OMM1415 OMY4140_ctg858
OMM1142 OmyFGT17TUF
59.8 Ssa0025(BHMS185)
68.4 BHMS429 OMM1796b
69.2 OMS00139
69.4 OMM3115_MHC1B
69.5 OMM1447 OMM5243
70.2 OMM3089_MHC1B
72.7 OMM1189 OMM1384a
OMY4191_ctg2776
75.1 OMM5192 Ogo1UW
OmyRGT41TUF OMYFGT5TUF
OMM5271
77.2 OMM3155_TLR1 OMM3154_TLR1
OMY4153_ctg1079
79.3 OMM1596
82.1 OMM1568 BHMS206
OMYRT2TUF
83.5 OMM1514 OMM5264
OMM1312
85.6 OMY4183a_ctg2417_or_?
86.4 Ogo2b
91.5 OMS00072
94.4 OMS00184
99.0 OMM1510 OMS00021
106.9 BHMS267
109.5 Oneu8 OMM1297
131.8 OMM5174

# Omy15

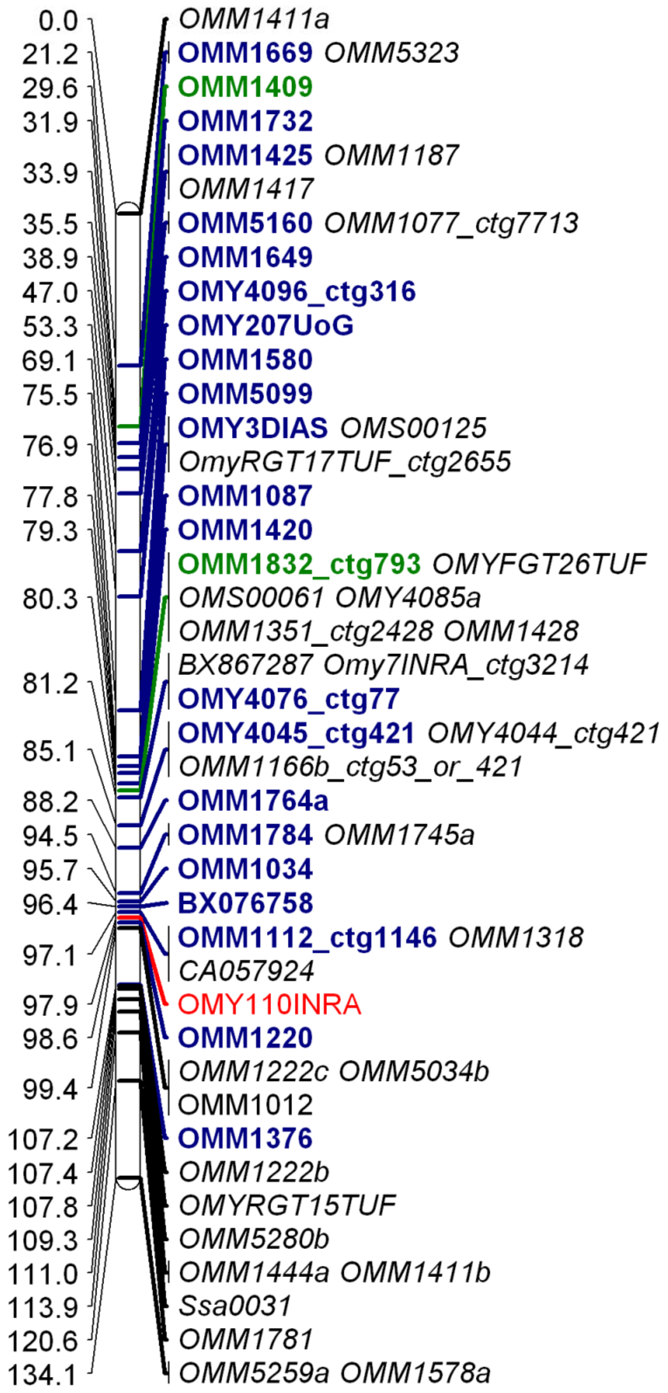
0.0 OMM1411a
21.2 OMM1669 OMM5323
29.6 OMM1409
31.9 OMM1732
33.9 OMM1425 OMM1187
OMM1417
35.5 OMM5160 OMM1077_ctg7713
38.9 OMM1649
47.0 OMY4096_ctg316
53.3 OMY207UoG
69.1 OMM1580
75.5 OMM5099
76.9 OMY3DIAS OMS00125
OmyRGT17TUF_ctg2655
77.8 OMM1087
79.3 OMM1420
80.3 OMM1832_ctg793 OMYFGT26TUF
OMS00061 OMY4085a
OMM1351_ctg2428 OMM1428
81.2 BX867287 Omy7INRA_ctg3214
OMY4076_ctg77
85.1 OMY4045_ctg421 OMY4044_ctg421
OMM1166b_ctg53_or_421
88.2 OMM1764a
94.5 OMM1784 OMM1745a
95.7 OMM1034
96.4 BX076758
97.1 OMM1112_ctg1146 OMM1318
CA057924
97.9 OMY110INRA
98.6 OMM1220
99.4 OMM1222c OMM5034b
OMM1012
107.2 OMM1376
107.4 OMM1222b
107.8 OMYRGT15TUF
109.3 OMM5280b
111.0 OMM1444a OMM1411b
113.9 Ssa0031
120.6 OMM1781
134.1 OMM5259a OMM1578a

# Omy16

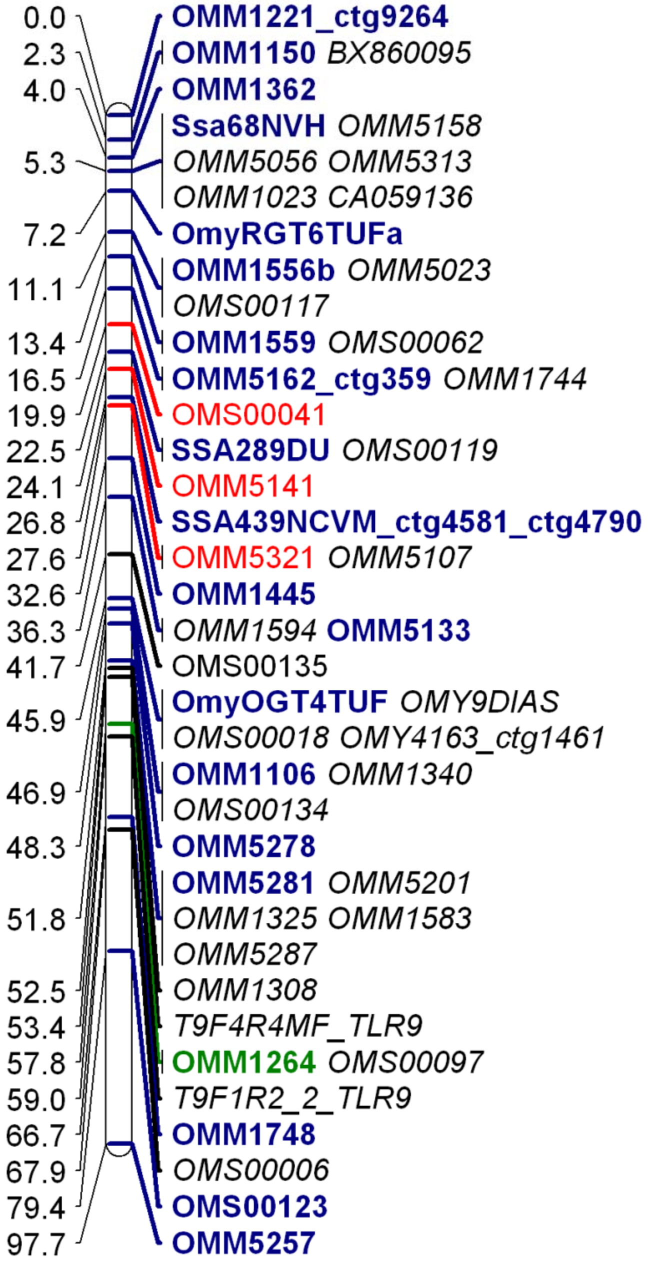
0.0 OMM1221_ctg9264
2.3 OMM1150 BX860095
4.0 OMM1362
Ssa68NVH OMM5158
5.3 OMM5056 OMM5313
OMM1023 CA059136
7.2 OmyRGT6TUFa
11.1 OMM1556b OMM5023
OMS00117
13.4 OMM1559 OMS00062
16.5 OMM5162_ctg359 OMM1744
19.9 OMS00041
22.5 SSA289DU OMS00119
24.1 OMM5141
26.8 SSA439NCVM_ctg4581_ctg4790
27.6 OMM5321 OMM5107
32.6 OMM1445
36.3 OMM1594 OMM5133
41.7 OMS00135
OmyOGT4TUF OMY9DIAS
45.9 OMS00018 OMY4163_ctg1461
OMM1106 OMM1340
46.9 OMS00134
48.3 OMM5278
OMM5281 OMM5201
51.8 OMM1325 OMM1583
OMM5287
52.5 OMM1308
53.4 T9F4R4MF_TLR9
57.8 OMM1264 OMS00097
59.0 T9F1R2_2_TLR9
66.7 OMM1748
67.9 OMS00006
79.4 OMS00123
97.7 OMM5257

# Omy17

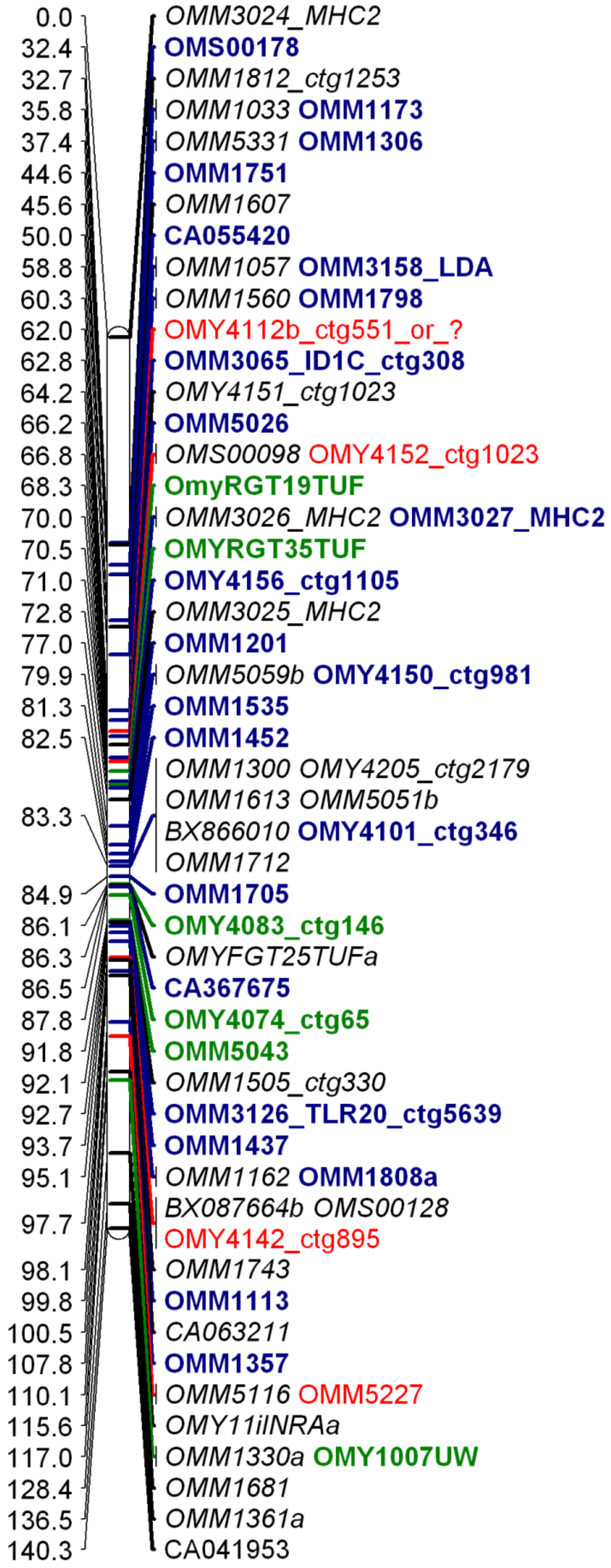

0.0 OMM3024_MHC2
32.4 OMS00178
32.7 OMM1812_ctg1253
35.8 OMM1033 OMM1173
37.4 OMM5331 OMM1306
44.6 OMM1751
45.6 OMM1607
50.0 CA055420
58.8 OMM1057 OMM3158_LDA
60.3 OMM1560 OMM1798
62.0 OMY4112b_ctg551_or_?
62.8 OMM3065_ID1C_ctg308
64.2 OMY4151_ctg1023
66.2 OMM5026
66.8 OMS00098 OMY4152_ctg1023
68.3 OmyRGT19TUF
70.0 OMM3026_MHC2 OMM3027_MHC2
70.5 OMYRGT35TUF
71.0 OMY4156_ctg1105
72.8 OMM3025_MHC2
77.0 OMM1201
79.9 OMM5059b OMY4150_ctg981
81.3 OMM1535
82.5 OMM1452
OMM1300 OMY4205_ctg2179
OMM1613 OMM5051b
83.3 BX866010 OMY4101_ctg346
OMM1712
84.9 OMM1705
86.1 OMY4083_ctg146
86.3 OMYFGT25TUFa
86.5 CA367675
87.8 OMY4074_ctg65
91.8 OMM5043
92.1 OMM1505_ctg330
92.7 OMM3126_TLR20_ctg5639
93.7 OMM1437
95.1 OMM1162 OMM1808a
97.7 BX087664b OMS00128
OMY4142_ctg895
98.1 OMM1743
99.8 OMM1113
100.5 CA063211
107.8 OMM1357
110.1 OMM5116 OMM5227
115.6 OMY11iINRAa
117.0 OMM1330a OMY1007UW
128.4 OMM1681
136.5 OMM1361a
140.3 CA041953

## Omy18

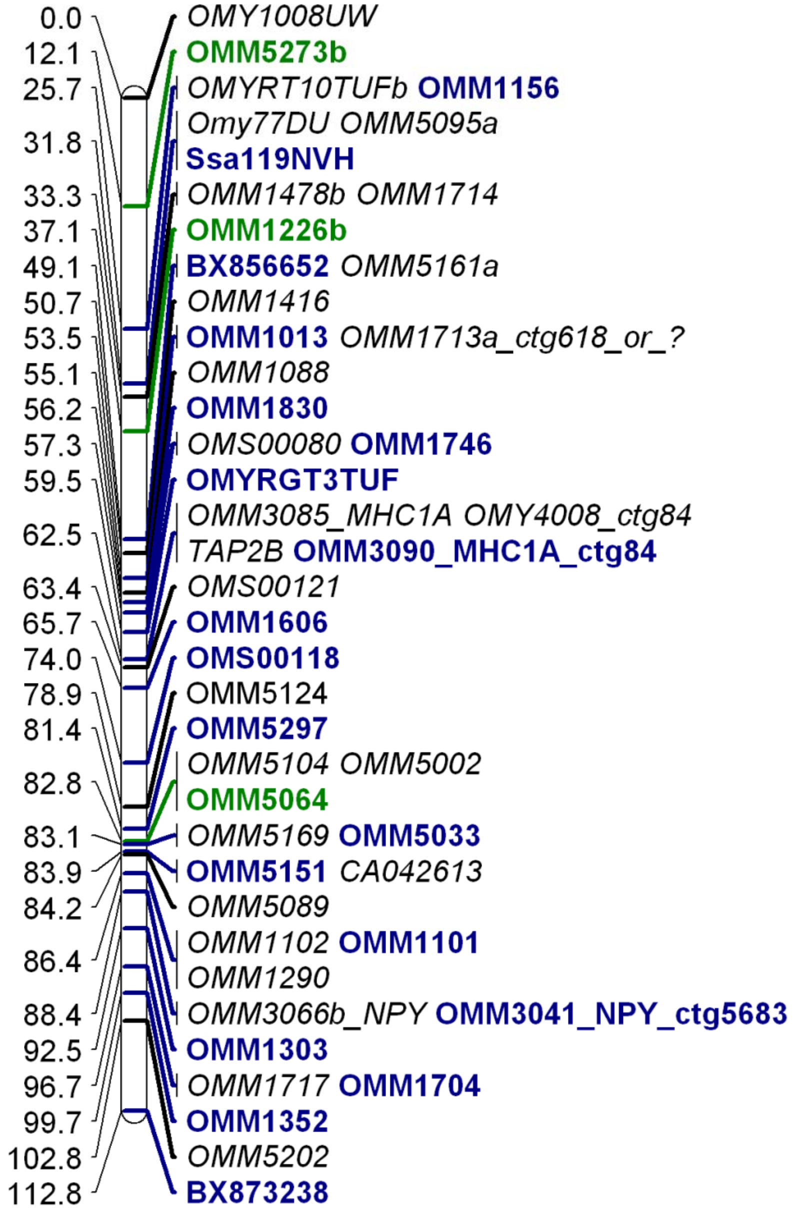
0.0
12.1
25.7
31.8
33.3
37.1
49.1
50.7
53.5
55.1
56.2
57.3
59.5
62.5
63.4
65.7
74.0
78.9
81.4
82.8
83.1
83.9
84.2
86.4
88.4
92.5
96.7
99.7
102.8
112.8
OMY1008UW
OMM5273b
OMYRT10TUFb OMM1156
Omy77DU OMM5095a
Ssa119NVH
OMM1478b OMM1714
OMM1226b
BX856652 OMM5161a
OMM1416
OMM1013 OMM1713a_ctg618_or_?
OMM1088
OMM1830
OMS00080 OMM1746
OMYRGT3TUF
OMM3085_MHC1A OMY4008_ctg84
TAP2B OMM3090_MHC1A_ctg84
OMS00121
OMM1606
OMS00118
OMM5124
OMM5297
OMM5104 OMM5002
OMM5064
OMM5169 OMM5033
OMM5151 CA042613
OMM5089
OMM1102 OMM1101
OMM1290
OMM3066b_NPY OMM3041_NPY_ctg5683
OMM1303
OMM1717 OMM1704
OMM1352
OMM5202
BX873238

## Omy19

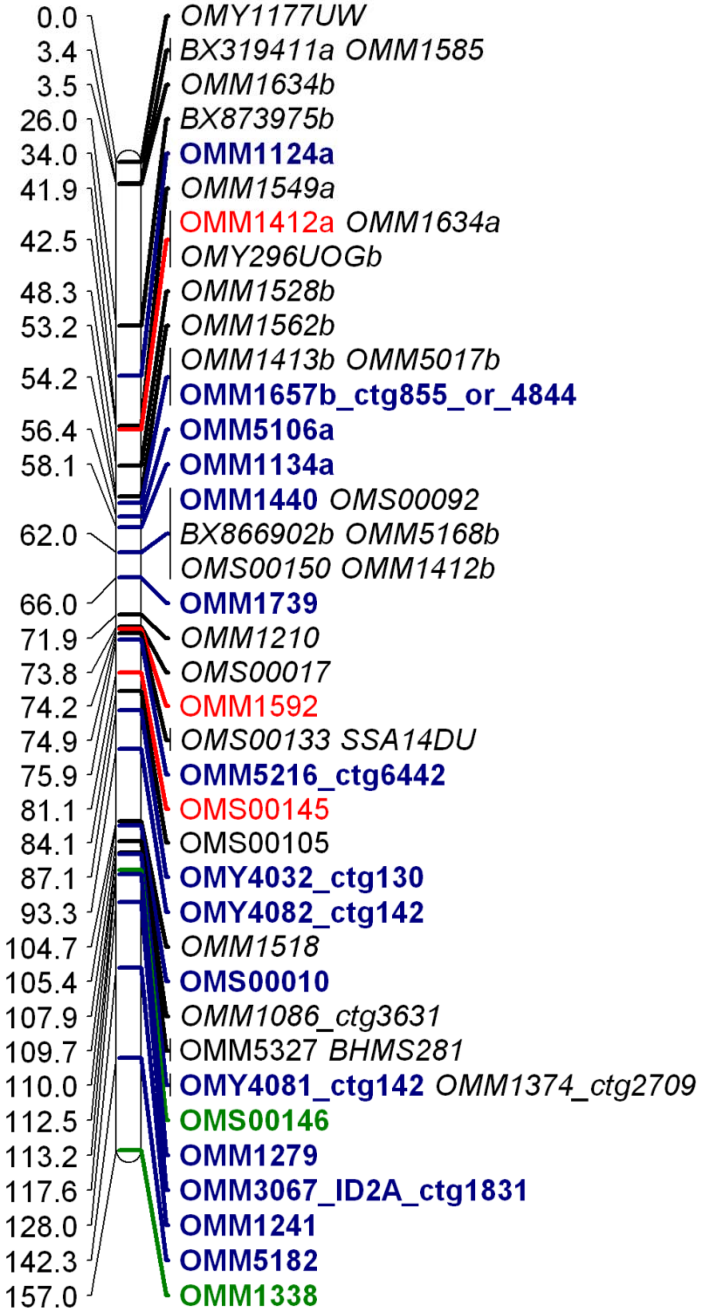
0.0
3.4
3.5
26.0
34.0
41.9
42.5
48.3
53.2
54.2
56.4
58.1
62.0
66.0
71.9
73.8
74.2
74.9
75.9
81.1
84.1
87.1
93.3
104.7
105.4
107.9
109.7
110.0
112.5
113.2
117.6
128.0
142.3
157.0
OMY1177UW
BX319411a OMM1585
OMM1634b
BX873975b
OMM1124a
OMM1549a
OMM1412a OMM1634a
OMY296UOGb
OMM1528b
OMM1562b
OMM1413b OMM5017b
OMM1657b_ctg855_or_4844
OMM5106a
OMM1134a
OMM1440 OMS00092
BX866902b OMM5168b
OMS00150 OMM1412b
OMM1739
OMM1210
OMS00017
OMM1592
OMS00133 SSA14DU
OMM5216_ctg6442
OMS00145
OMS00105
OMY4032_ctg130
OMY4082_ctg142
OMM1518
OMS00010
OMM1086_ctg3631
OMM5327 BHMS281
OMY4081_ctg142 OMM1374_ctg2709
OMS00146
OMM1279
OMM3067_ID2A_ctg1831
OMM1241
OMM5182
OMM1338

## Omy20

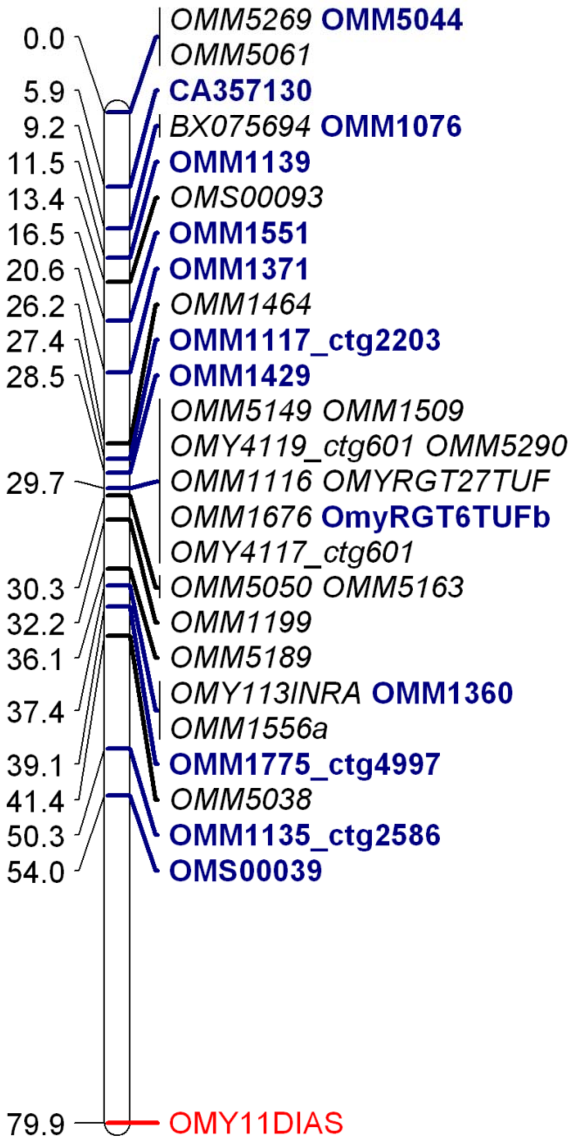

0.0
5.9
9.2
11.5
13.4
16.5
20.6
26.2
27.4
28.5
29.7
30.3
32.2
36.1
37.4
39.1
41.4
50.3
54.0
79.9
OMM5269 OMM5044
OMM5061
CA357130
BX075694 OMM1076
OMM1139
OMS00093
OMM1551
OMM1371
OMM1464
OMM1117_ctg2203
OMM1429
OMM5149 OMM1509
OMY4119_ctg601 OMM5290
OMM1116 OMYRGT27TUF
OMM1676 OmyRGT6TUFb
OMY4117_ctg601
OMM5050 OMM5163
OMM1199
OMM5189
OMY113INRA OMM1360
OMM1556a
OMM1775_ctg4997
OMM5038
OMM1135_ctg2586
OMS00039
OMY11DIAS

# Omy21

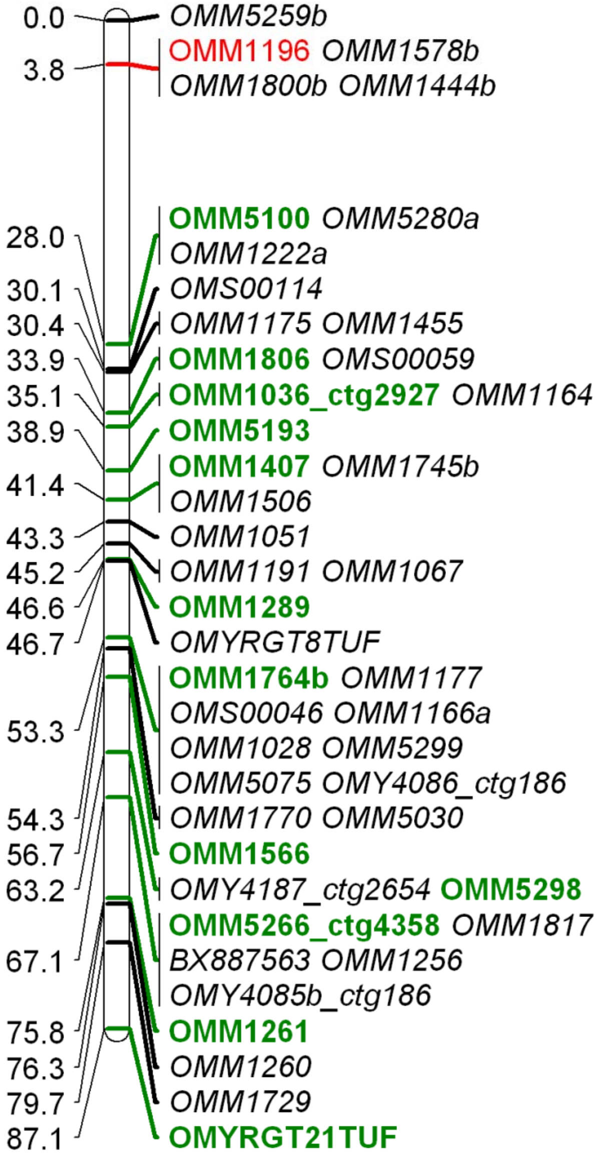
0.0
3.8
28.0
30.1
30.4
33.9
35.1
38.9
41.4
43.3
45.2
46.6
46.7
53.3
54.3
56.7
63.2
67.1
75.8
76.3
79.7
87.1
OMM5259b
OMM1196 OMM1578b
OMM1800b OMM1444b
OMM5100 OMM5280a
OMM1222a
OMS00114
OMM1175 OMM1455
OMM1806 OMS00059
OMM1036_ctg2927 OMM1164
OMM5193
OMM1407 OMM1745b
OMM1506
OMM1051
OMM1191 OMM1067
OMM1289
OMYRGT8TUF
OMM1764b OMM1177
OMS00046 OMM1166a
OMM1028 OMM5299
OMM5075 OMY4086_ctg186
OMM1770 OMM5030
OMM1566
OMY4187_ctg2654 OMM5298
OMM5266_ctg4358 OMM1817
BX887563 OMM1256
OMY4085b_ctg186
OMM1261
OMM1260
OMM1729
OMYRGT21TUF

## Omy22

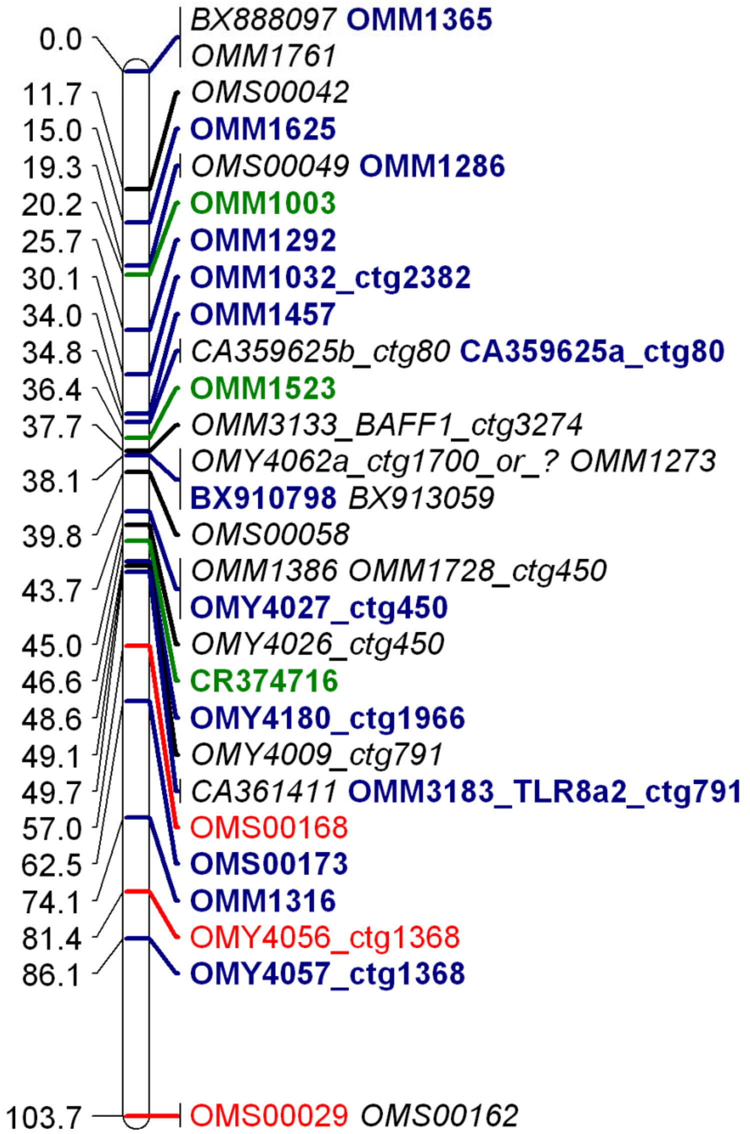

0.0
11.7
15.0
19.3
20.2
25.7
30.1
34.0
34.8
36.4
37.7
38.1
39.8
43.7
45.0
46.6
48.6
49.1
49.7
57.0
62.5
74.1
81.4
86.1
103.7
BX888097 OMM1365
OMM1761
OMS00042
OMM1625
OMS00049 OMM1286
OMM1003
OMM1292
OMM1032_ctg2382
OMM1457
CA359625b_ctg80 CA359625a_ctg80
OMM1523
OMM3133_BAFF1_ctg3274
OMY4062a_ctg1700_or_? OMM1273
BX910798 BX913059
OMS00058
OMM1386 OMM1728_ctg450
OMY4027_ctg450
OMY4026_ctg450
CR374716
OMY4180_ctg1966
OMY4009_ctg791
CA361411 OMM3183_TLR8a2_ctg791
OMS00168
OMS00173
OMM1316
OMY4056_ctg1368
OMY4057_ctg1368
OMS00029 OMS00162

## Omy23

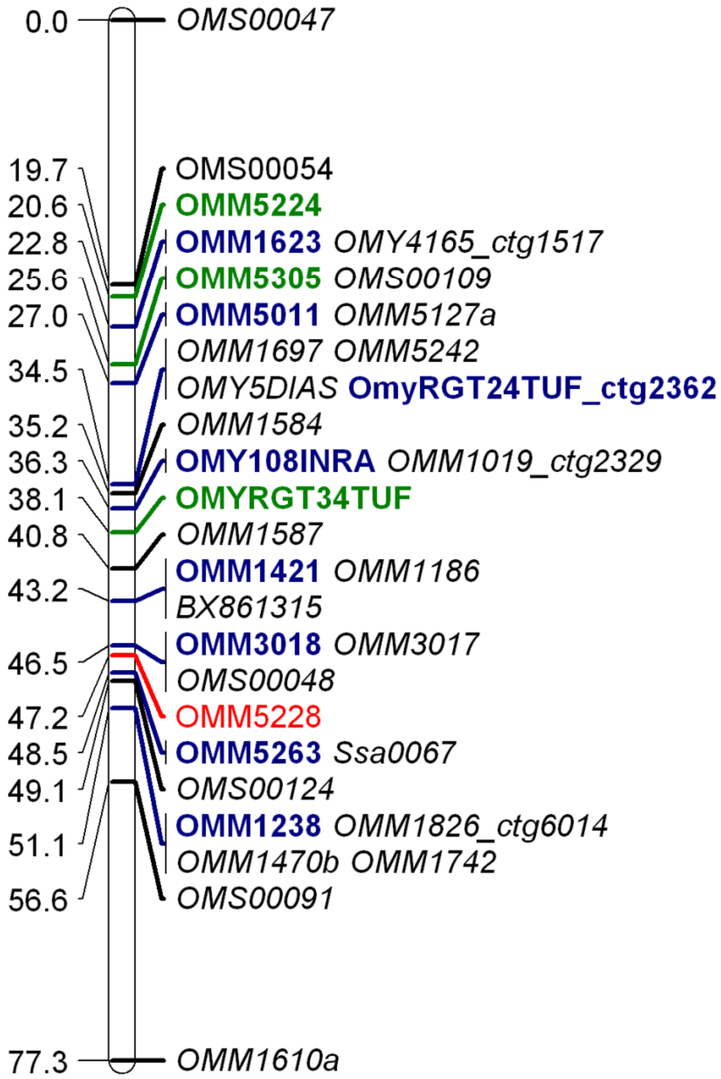
0.0
OMS00047
19.7
OMS00054
20.6
OMM5224
22.8
OMM1623 OMY4165_ctg1517
25.6
OMM5305 OMS00109
27.0
OMM5011 OMM5127a
OMM1697 OMM5242
34.5
OMY5DIAS OmyRGT24TUF_ctg2362
35.2
OMM1584
36.3
OMY108INRA OMM1019_ctg2329
38.1
OMYRGT34TUF
40.8
OMM1587
OMM1421 OMM1186
43.2
BX861315
OMM3018 OMM3017
46.5
OMS00048
47.2
OMM5228
48.5
OMM5263 Ssa0067
49.1
OMS00124
OMM1238 OMM1826_ctg6014
51.1
OMM1470b OMM1742
56.6
OMS00091
77.3
OMM1610a

## Omy24

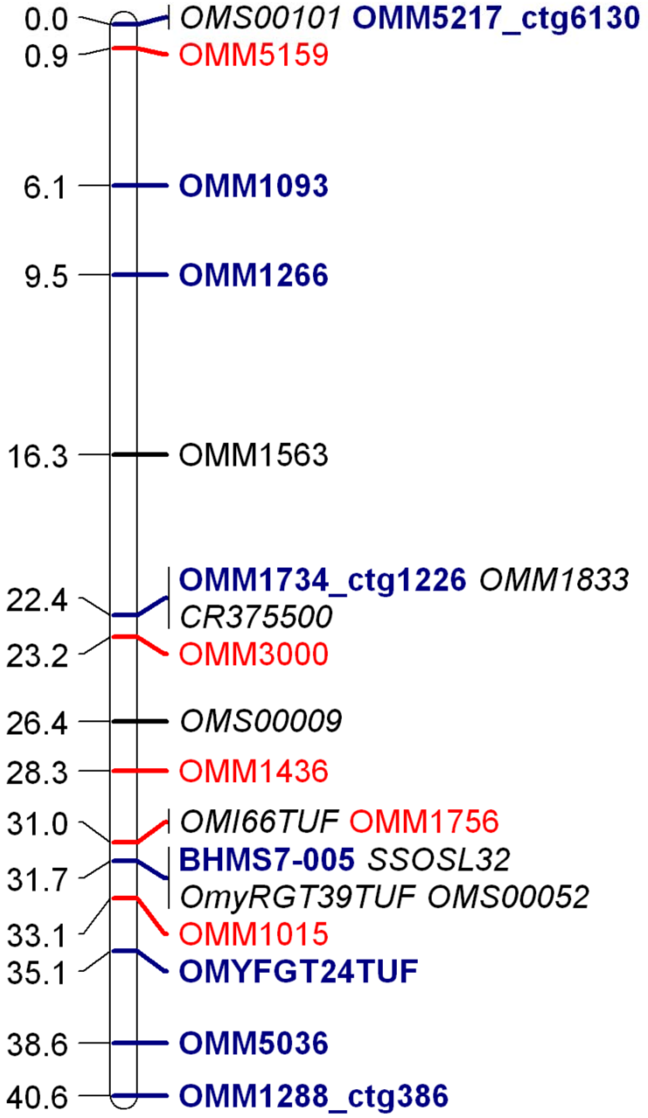
0.0 OMS00101 OMM5217_ctg6130
0.9 OMM5159
6.1 OMM1093
9.5 OMM1266
16.3 OMM1563
22.4 OMM1734_ctg1226 OMM1833
CR375500
23.2 OMM3000
26.4 OMS00009
28.3 OMM1436
31.0 OMI66TUF OMM1756
31.7 BHMS7-005 SSOSL32
OmyRGT39TUF OMS00052
33.1 OMM1015
35.1 OMYFGT24TUF
38.6 OMM5036
40.6 OMM1288_ctg386

## Omy25

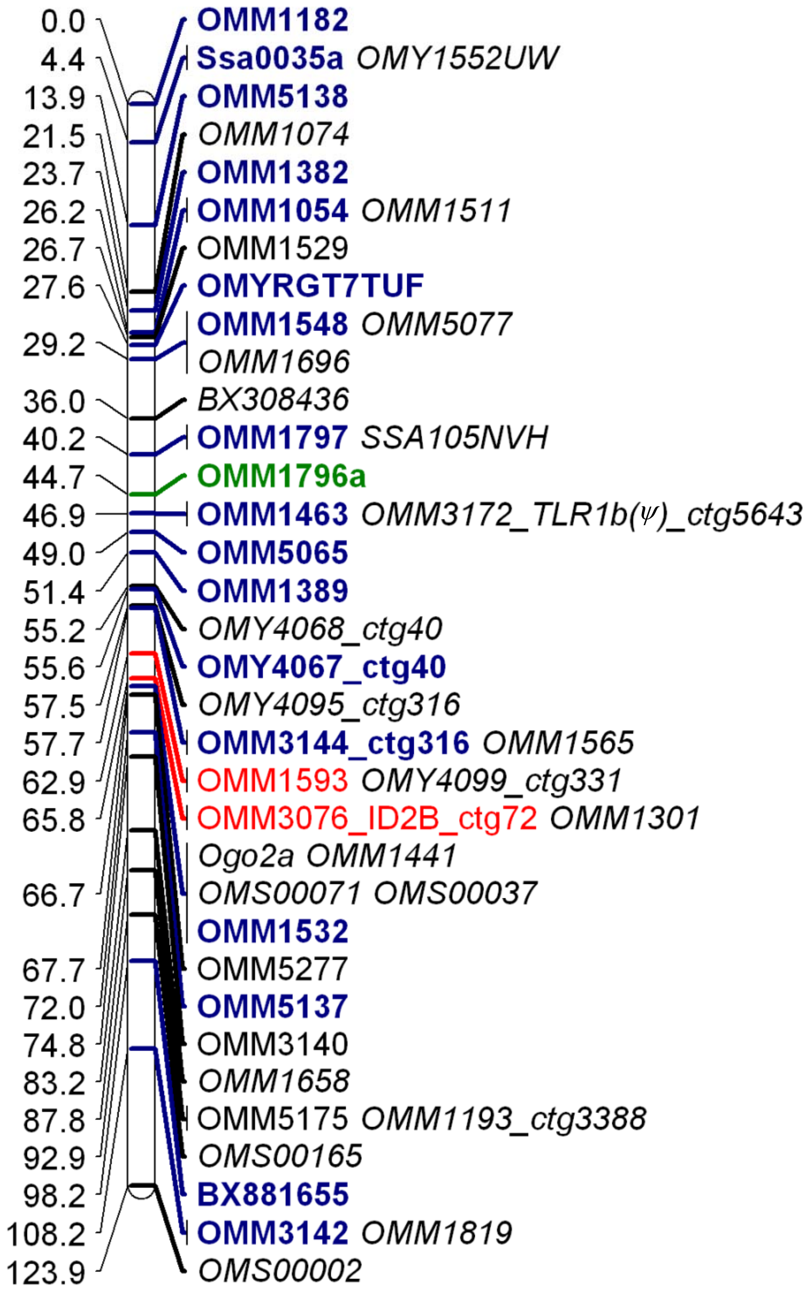

0.0 OMM1182
4.4 Ssa0035a OMY1552UW
13.9 OMM5138
21.5 OMM1074
23.7 OMM1382
26.2 OMM1054 OMM1511
26.7 OMM1529
27.6 OMYRGT7TUF
29.2 OMM1548 OMM5077
OMM1696
36.0 BX308436
40.2 OMM1797 SSA105NVH
44.7 OMM1796a
46.9 OMM1463 OMM3172_TLR1b(ψ)_ctg5643
49.0 OMM5065
51.4 OMM1389
55.2 OMY4068_ctg40
55.6 OMY4067_ctg40
57.5 OMY4095_ctg316
57.7 OMM3144_ctg316 OMM1565
62.9 OMM1593 OMY4099_ctg331
65.8 OMM3076_ID2B_ctg72 OMM1301
Ogo2a OMM1441
66.7 OMS00071 OMS00037
OMM1532
67.7 OMM5277
72.0 OMM5137
74.8 OMM3140
83.2 OMM1658
87.8 OMM5175 OMM1193_ctg3388
92.9 OMS00165
98.2 BX881655
108.2 OMM3142 OMM1819
123.9 OMS00002

# Omy26

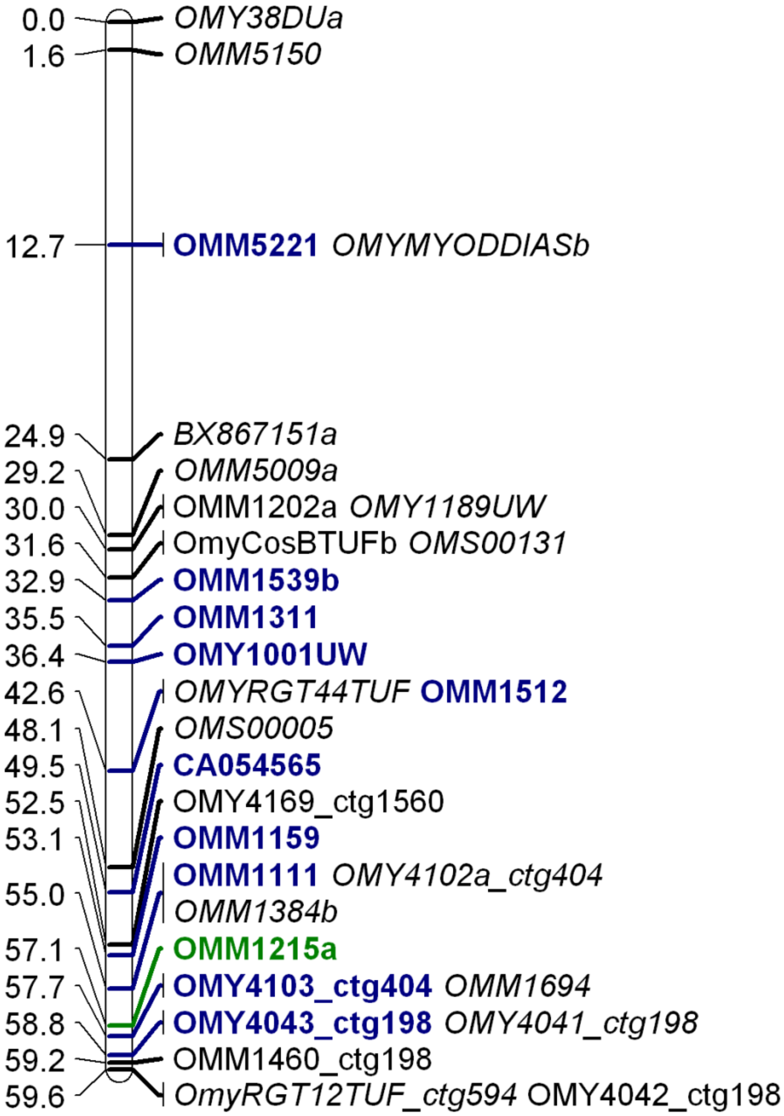

0.0 OMY38DUa
1.6 OMM5150
12.7 OMM5221 OMYMYODDIASb
24.9 BX867151a
29.2 OMM5009a
30.0 OMM1202a OMY1189UW
31.6 OmyCosBTUFb OMS00131
32.9 OMM1539b
35.5 OMM1311
36.4 OMY1001UW
42.6 OMYRGT44TUF OMM1512
48.1 OMS00005
49.5 CA054565
52.5 OMY4169_ctg1560
53.1 OMM1159
OMM1111 OMY4102a_ctg404
55.0 OMM1384b
57.1 OMM1215a
57.7 OMY4103_ctg404 OMM1694
58.8 OMY4043_ctg198 OMY4041_ctg198
59.2 OMM1460_ctg198
59.6 OmyRGT12TUF_ctg594 OMY4042_ctg198

## Omy27

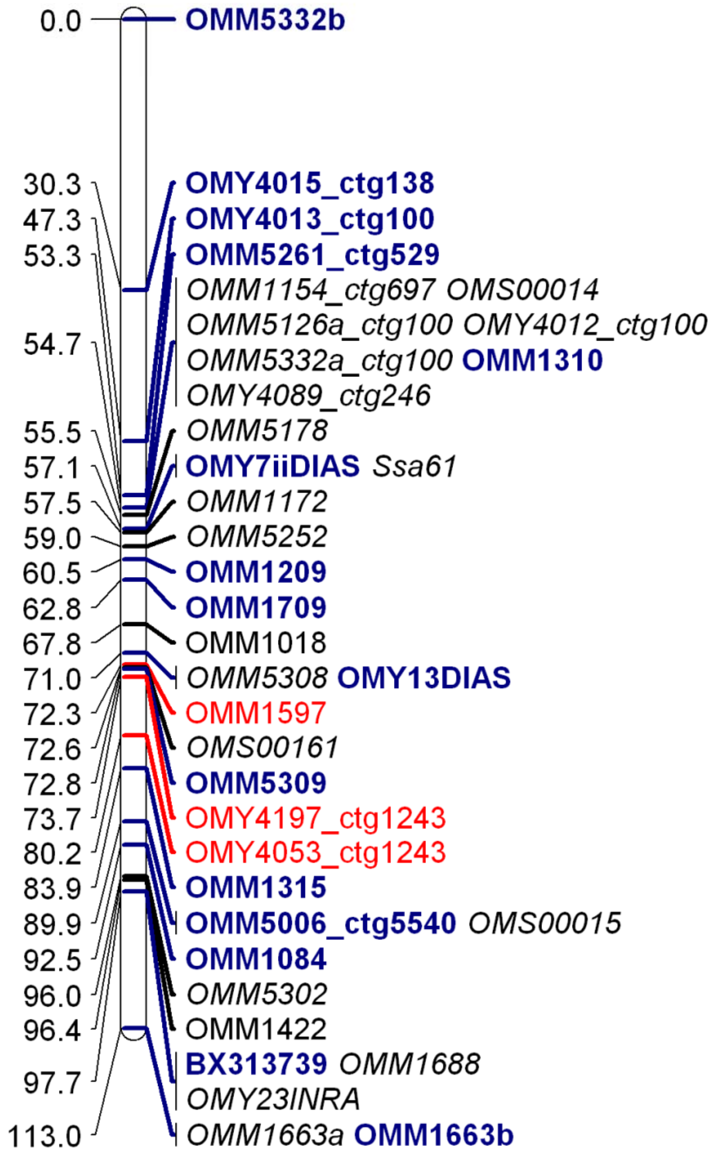
0.0 OMM5332b
30.3 OMY4015_ctg138
47.3 OMY4013_ctg100
53.3 OMM5261_ctg529
OMM1154_ctg697 OMS00014
54.7 OMM5126a_ctg100 OMY4012_ctg100
OMM5332a_ctg100 OMM1310
OMY4089_ctg246
55.5 OMM5178
57.1 OMY7iiDIAS Ssa61
57.5 OMM1172
59.0 OMM5252
60.5 OMM1209
62.8 OMM1709
67.8 OMM1018
71.0 OMM5308 OMY13DIAS
72.3 OMM1597
72.6 OMS00161
72.8 OMM5309
73.7 OMY4197_ctg1243
80.2 OMY4053_ctg1243
83.9 OMM1315
89.9 OMM5006_ctg5540 OMS00015
92.5 OMM1084
96.0 OMM5302
96.4 OMM1422
BX313739 OMM1688
97.7 OMY23INRA
113.0 OMM1663a OMM1663b

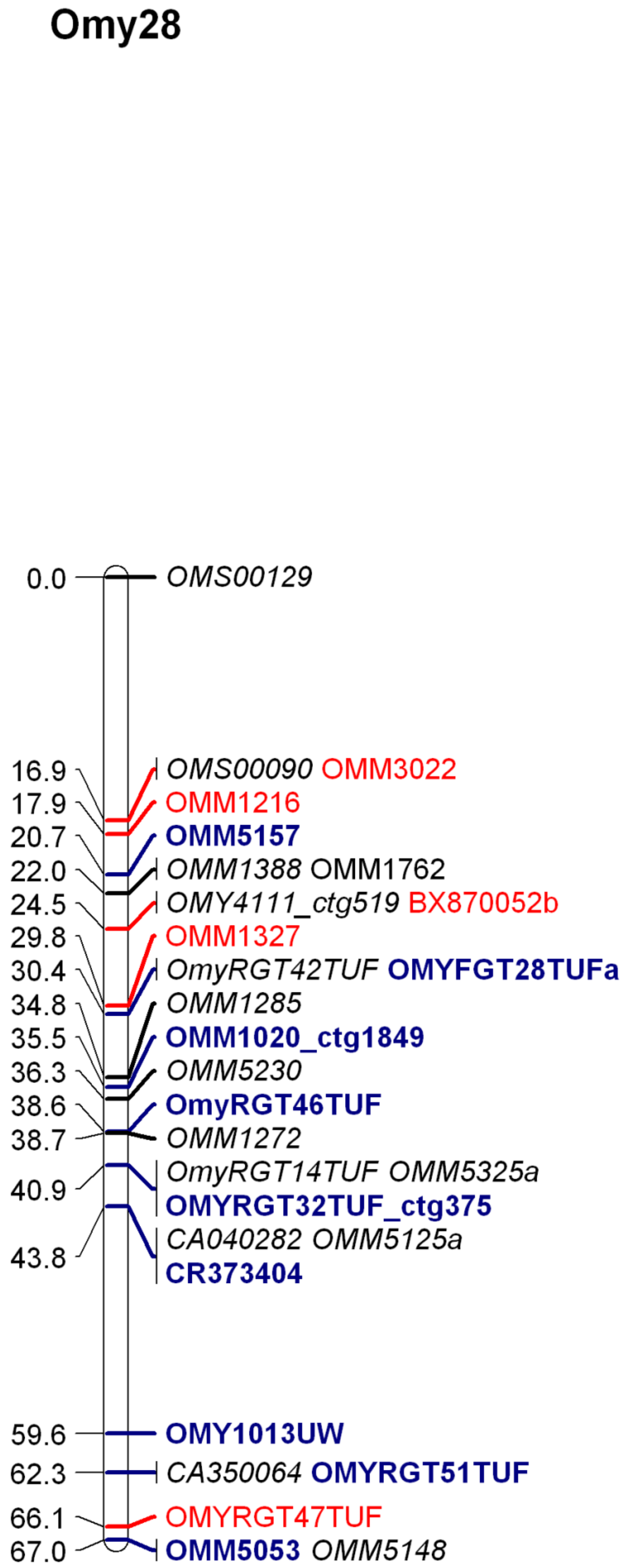

Omy28
0.0 OMS00129
16.9 OMS00090 OMM3022
17.9 OMM1216
20.7 OMM5157
22.0 OMM1388 OMM1762
24.5 OMY4111_ctg519 BX870052b
29.8 OMM1327
30.4 OmyRGT42TUF OMYFGT28TUFa
34.8 OMM1285
35.5 OMM1020_ctg1849
36.3 OMM5230
38.6 OmyRGT46TUF
38.7 OMM1272
OmyRGT14TUF OMM5325a
40.9 OMYRGT32TUF_ctg375
CA040282 OMM5125a
43.8 CR373404
59.6 OMY1013UW
62.3 CA350064 OMYRGT51TUF
66.1 OMYRGT47TUF
67.0 OMM5053 OMM5148

# OmySex

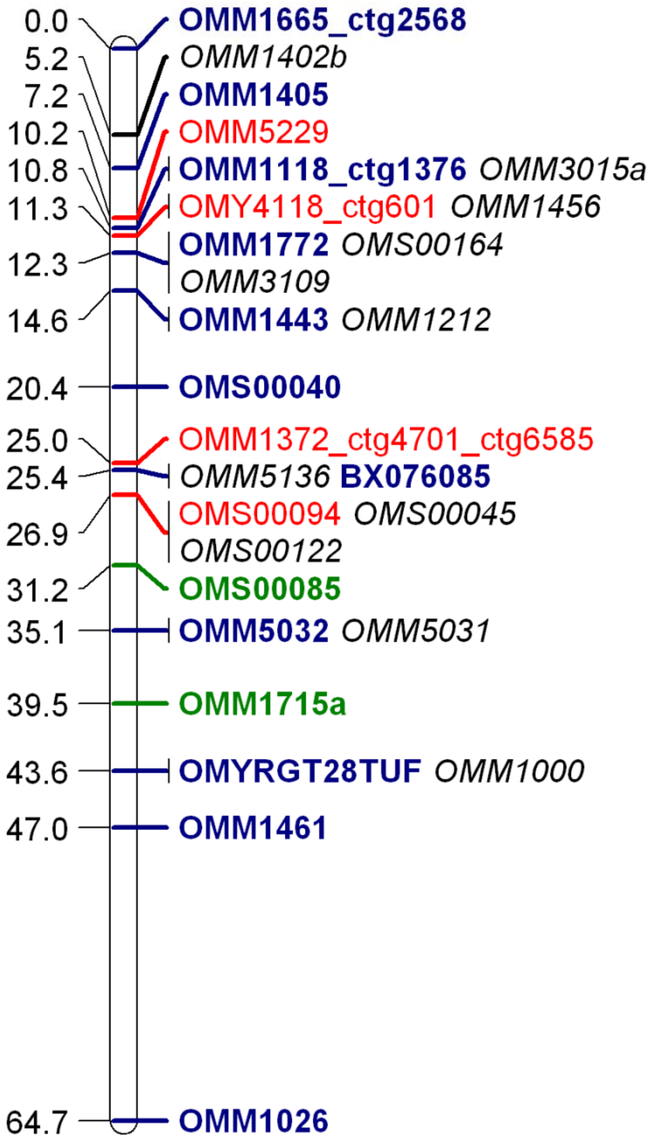
0.0
5.2
7.2
10.2
10.8
11.3
12.3
14.6
20.4
25.0
25.4
26.9
31.2
35.1
39.5
43.6
47.0
64.7
OMM1665_ctg2568
OMM1402b
OMM1405
OMM5229
OMM1118_ctg1376 OMM3015a
OMY4118_ctg601 OMM1456
OMM1772 OMS00164
OMM3109
OMM1443 OMM1212
OMS00040
OMM1372_ctg4701_ctg6585
OMM5136 BX076085
OMS00094 OMS00045
OMS00122
OMS00085
OMM5032 OMM5031
OMM1715a
OMYRGT28TUF OMM1000
OMM1461
OMM1026
